# Bio-Based Hemostatic Adhesives, Sealants, and Local Hemostatic Agents for Liver Tissue: A Systematic Review with Meta-Analysis of Preclinical Data

**DOI:** 10.3390/jfb17100511

**Published:** 2026-10-11

**Authors:** Roman Ishchenko, Maksim Solopov, Matvey Ilyashenko, Viktor Turchin, Dmitry Filimonov, Tatiana Kryuk, Anna Kavelina, Gulnara Akopian, Viktoria Bushe, Elizaveta Lebed, Andrey Popandopulo

**Affiliations:** 1 V.K. Gusak Institute of Emergency and Reconstructive Surgery, 283045 Donetsk, Russia; 2 Faculty of General Medicine No. 2, Gorky Donetsk National Medical University, 283003 Donetsk, Russia; 3 Litvinenko Institute of Physical-Organic and Coal Chemistry, 283048 Donetsk, Russia

**Keywords:** bioadhesives, tissue sealants, hemostasis, liver surgery, hepatectomy, biomaterials, translational gap, systematic review, meta-analysis

## Abstract

Background: Liver surgery carries a high risk of hemorrhage; bio-based adhesives and sealants are promising adjuncts, but hepatic evidence remains fragmented. Methods: Following PRISMA 2020, we searched PubMed, Dimensions, Consensus, and SciSpace through February 2026. Forty-two studies were included (38 preclinical animal, 3 human clinical, and 1 veterinary RCT); 25 preclinical studies (67 pairwise comparisons) underwent random-effects meta-analysis using REML with HKSJ adjustment. Risk of bias was assessed with SYRCLE, RoB 2, and ROBINS-I; certainty of evidence was rated with an adapted GRADE framework. Results: Versus standard hemostats, bioadhesives shortened time to hemostasis (MD = +56.05 s, 95% CI: 20.14 to 91.96; *p* = 0.0044) and reduced blood loss (RoM = 2.05, 95% CI: 1.48 to 2.86; *p* = 0.0002); versus untreated negative controls, effects were larger (MD = +419.53 s; RoM = 5.68). Heterogeneity was extreme (*I*^2^ = 96–99%), 95% prediction intervals included the null, and available clinical effect sizes were small and non-significant. Certainty of evidence was low to very low. Conclusions: Bio-based adhesives show substantial preclinical hemostatic effects in liver models, but these findings should not be interpreted as established clinical benefit. Adequately powered multicenter RCTs with standardized outcome assessment are required.

## 1. Introduction

Massive uncontrolled hemorrhage remains a leading cause of trauma-associated morbidity and mortality and poses a major challenge during extensive surgical resection of solid parenchymal organs [1,2]. The liver’s distinctive anatomical and histological architecture—characterized by marked friability, the absence of contractile elements around intrahepatic vessels, and extensive sinusoidal vascularization—makes intraoperative hemostasis particularly challenging [3,4]. Conventional physical and mechanical techniques, including monopolar and bipolar electrocautery, parenchymal suturing, and gauze packing, may be suboptimal. Parenchymal sutures can cut through fragile liver tissue, whereas standard packing requires prolonged manual compression and may provide inadequate control of profuse parenchymal bleeding in trauma- or hemodilution-induced coagulopathy [5,6].

Hemostatic control is particularly challenging during extensive liver resections, in which temporary vascular inflow occlusion, including the Pringle maneuver, is commonly used to limit blood loss [7]. Although inflow occlusion facilitates parenchymal transection, it inevitably induces warm hepatic ischemia, and subsequent reperfusion contributes to the pathophysiological cascade of ischemia–reperfusion injury (IRI) [8]. Hemorrhage control and limitation of ischemic parenchymal injury are therefore interrelated clinical objectives: topical hemostatic agents may serve as useful adjuncts to vascular occlusion and potentially reduce the duration or frequency of inflow-clamping cycles.

In this context, there is a compelling clinical need to develop bioadhesives and tissue sealants capable of rapid, leak-tight *in situ* sealing of visceral wounds [9,10]. In contrast to fully synthetic surgical glues such as cyanoacrylates, which can cause marked local cytotoxicity and foreign-body reactions and can form rigid barriers that impede tissue regeneration, adhesives derived from biological polymers generally exhibit favorable cytocompatibility, low cytotoxicity, and susceptibility to physiological enzymatic biodegradation [11,12].

The potential clinical utility of bioadhesives and tissue sealants in hepatopancreatobiliary surgery extends well beyond passive mechanical hemostasis. Atraumatic local hemostasis can reduce the need for repeated electrocoagulation, deep parenchymal transfixion sutures, and aggressive compression, thereby limiting secondary thermal and mechanical tissue injury and the associated release of damage-associated molecular patterns (DAMPs) [13]. Furthermore, prompt hemostatic sealing may reduce hypovolemia, visceral hypoperfusion, and allogeneic blood transfusion requirements—factors known to exacerbate the systemic inflammatory response during reperfusion [14].

Historically, fibrin- and collagen-based sealants that mimic the terminal stage of the physiological coagulation cascade have achieved the widest clinical adoption [15,16]. Despite the demonstrated clinical safety of commercial products such as TachoSil and investigational fibrin films such as sFilm-FS [17,18], their use may be limited by modest shear adhesion on profusely wet, bleeding, and dynamically moving visceral surfaces [19,20]. To address these limitations, modified collagen- and gelatin-based matrices have been developed, including photocrosslinkable gelatin methacryloyl (GelMA) and catechol-functionalized gelatin methacryloyl (GelMAC), which form strong covalent interfacial bonds with nucleophilic amino groups in tissue proteins and can withstand high burst pressures [21,22,23].

In parallel, substantial biomaterials research has focused on polysaccharide-based hemostatic platforms, particularly alginate and chitosan [24,25]. Chitosan has been extensively investigated because its polycationic charge promotes erythrocyte aggregation and platelet adhesion through electrostatic interactions, thereby facilitating rapid formation of a stable physical clot even in the absence of endogenous coagulation factors [26,27]. Advanced architectures, including microneedle patch arrays [28,29], nanostructured aerogels [30], and lyophilized cryogels [31,32], enable these biomaterials to rapidly absorb whole blood, concentrate clotting components, and mechanically interlock with the parenchymal microvasculature.

Despite the rapid growth of research on novel hemostatic biomaterials, evidence regarding their comparative efficacy, mechanical performance, and safety remains highly fragmented. To date, no rigorous, methodologically standardized appraisal of this preclinical literature has been conducted from an evidence-based perspective. Therefore, the primary objective of this systematic review and meta-analysis was to synthesize and, where methodologically appropriate, quantitatively pool evidence on the hemostatic efficacy, mechanical properties, and biocompatibility of bio-based adhesives, sealants, and topical hemostats for liver tissue; to assess their potential role in mitigating surgical and ischemia–reperfusion injury; to appraise the certainty of the evidence; and to identify key translational barriers to clinical adoption.

## 2. Materials and Methods

### 2.1. Protocol and Registration

This systematic review was conducted and reported in accordance with the Preferred Reporting Items for Systematic Reviews and Meta-Analyses (PRISMA 2020) statement [33]. The protocol is provided in the Supplementary Materials (Document S1). The protocol was registered retrospectively on the Open Science Framework platform (https://osf.io/kmvgp [accessed on 7 October 2026]). The core research question was structured using the Population, Intervention, Comparator, and Outcomes (PICO) framework (Table 1) [34].

To avoid overlap in the operational and chemical classification of biologically derived interventions and comparators (e.g., commercial collagen sponges or oxidized cellulose), comparators were strictly defined as established commercial standard-of-care products or conventional surgical controls, whereas interventions were defined as novel investigational bioadhesive platforms, irrespective of whether the base polymer belonged to the same biochemical class.

### 2.2. Information Sources and Search Strategy

A systematic literature search was conducted in the electronic bibliographic databases PubMed and Dimensions and using the scholarly search engines Consensus and SciSpace, without publication-date restrictions, through February 2026. The search strategy combined three Boolean concept blocks: (1) anatomical target (liver, hepatic, hepatectomy); (2) biomaterial class (adhesive, glue, sealant, hemostat, fibrin, gelatin, collagen, chitosan, alginate, hydrogel, cellulose, etc.); and (3) outcomes of interest (hemostasis, sealing, burst pressure, adhesion strength, shear strength, blood loss, biocompatibility, etc.). Because institutional subscription access to closed commercial databases (Embase, Scopus, Web of Science) was unavailable, the search was restricted to open or publicly accessible resources; this restriction is acknowledged as a methodological limitation. Comprehensive search strings for all platforms are provided in the Supplementary Materials.

### 2.3. Study Selection and Data Extraction

After automated deduplication, two reviewers independently screened titles and abstracts against predefined eligibility criteria and then independently assessed full texts. Disagreements were resolved by consensus or, when necessary, adjudication by a third senior reviewer. Data were extracted using a standardized, piloted form that captured general bibliographic information (study ID, first author, publication year, journal, DOI), experimental model characteristics (animal species, strain, weight, injury model, transection geometry), bioadhesive characteristics (polymeric base, chemical crosslinking, delivery format, application method), control groups, and primary and secondary outcomes (sample size per group, mean, and standard deviation [SD]). For studies reporting outcome data only graphically, numerical values were digitized using WebPlotDigitizer (v4.6) and flagged accordingly.

### 2.4. Risk-of-Bias and Certainty-of-Evidence Assessment

Methodological quality and risk of bias were assessed according to study design: (1) preclinical in vivo animal experiments were evaluated using the Systematic Review Centre for Laboratory Animal Experimentation (SYRCLE) risk-of-bias tool across 10 methodological domains; (2) randomized human clinical trials were assessed using the Cochrane Risk of Bias 2 (RoB 2) tool, whereas non-randomized cohorts were evaluated using the Risk Of Bias In Non-randomized Studies of Interventions (ROBINS-I) instrument; and (3) in vitro mechanical and cytotoxicity experiments were evaluated using a customized checklist assessing standardization of specimen dimensions, test velocity, environmental hydration, and calibration.

The overall certainty of the evidence for key outcomes was assessed using the Grading of Recommendations Assessment, Development, and Evaluation (GRADE) approach for clinical findings and its validated adaptation for preclinical animal intervention studies (SYRCLE framework), considering five core domains: risk of bias, inconsistency, indirectness, imprecision, and publication bias.

### 2.5. Data Synthesis and Statistical Analysis

Given the substantial clinical and methodological diversity across polymer formulations and surgical models, narrative synthesis was the primary approach across four outcome domains: hemostasis, mechanical properties, biocompatibility, and clinical outcomes. Quantitative meta-analysis was performed for the two continuous outcomes reported most consistently: time to hemostasis (TTH) and intraoperative blood loss (BL). In vitro mechanical properties (shear bond strength, tensile strength, and burst pressure) were synthesized descriptively because they represent intrinsic material characteristics, often lack paired intervention-versus-control comparators, and were measured using heterogeneous, non-standardized biomechanical assays.

#### 2.5.1. Population Separation and PICO Frameworks

To minimize biological confounding, quantitative meta-analysis was restricted to preclinical in vivo animal models (25 studies with complete continuous data, 67 comparisons). Human clinical trials (*k* = 3) and the veterinary clinical trial in client-owned dogs (*k* = 1) were analyzed separately using narrative and tabular synthesis, because vascular dynamics, coagulation pathways, hemorrhage rates, and transection dimensions differ substantially between laboratory rodents or swine and humans.

#### 2.5.2. Stratification by Material Class

To account for differences in polymer chemistry, interventions were stratified a priori into three classes according to the primary matrix polymer: (1) protein-based systems (fibrin, collagen, gelatin, peptide hydrogels); (2) polysaccharide-based systems (chitosan, alginate, oxidized cellulose, hyaluronic acid, pectin, starch, dextran); and (3) composite/chemically modified systems. Between-subgroup differences were examined using random-effects meta-regression with the Knapp-Hartung F-test.

#### 2.5.3. Stratification by Hemostatic Mechanism

In addition to chemical classification, each preclinical intervention (*n* = 25) was assigned to a primary functional hemostatic mechanism based on composition, physical form, and stated mode of action: (1) chemical tissue adhesion (catechol/dopamine-functionalized polymers, Schiff-base crosslinking, photoactivated hydrogels); (2) physical absorption and mechanical barrier formation (macroporous sponges, electrospun nanofiber meshes, cryogels, microneedle arrays, aerogels); (3) direct coagulation activation (topical thrombin, autologous platelet-fibrin complexes, procoagulant enzymes); and (4) combined mechanisms integrating at least two synergistic pathways.

#### 2.5.4. Effect Measures

The primary effect measures were (1) the raw mean difference (MD, seconds) for time to hemostasis, oriented so that MD > 0 indicates a shorter time to hemostasis with the bioadhesive, and (2) the ratio of means (RoM), calculated as the control-group mean divided by the intervention-group mean, for blood loss and time to hemostasis, where RoM > 1 indicates a reduction in blood loss or time favoring the bioadhesive. The standardized mean difference (Hedges’ *g*) was used only as a secondary sensitivity measure.

#### 2.5.5. Pooling Model

Meta-analysis was performed using random-effects models. Between-study variance (*τ^2^*) was estimated using restricted maximum likelihood (REML), and confidence intervals (CIs) and hypothesis tests were based on the Hartung–Knapp–Sidik–Jonkman (HKSJ) method (t-distribution with *k* − 1 degrees of freedom). The more conservative of the HKSJ and Wald standard errors was retained. This approach was selected instead of the traditional DerSimonian–Laird (DL) method because DL can underestimate between-study variance in the presence of extreme heterogeneity, producing overly narrow CIs and inflated type I error rates. Statistical heterogeneity was quantified using Cochran’s Q test, the *I*^2^ statistic, and *τ^2^*; 95% prediction intervals (PIs) were calculated to estimate the range of effects expected in future studies.

#### 2.5.6. Sources of Heterogeneity

Potential moderators of between-study variance were investigated using mixed-effects meta-regression with two prespecified covariates: (1) animal species (rodents vs. large animals/other) and (2) hepatic injury geometry (parenchymal resection vs. localized punch/puncture/incision). Moderator significance was assessed using the omnibus test of moderators (*Q_M_* and corresponding *p* value) and the proportion of explained between-study variance (*R*^2^).

#### 2.5.7. Sensitivity Analyses

Robustness was assessed using three predefined procedures: (1) exclusion of studies at high risk of bias in any key SYRCLE domain; (2) restriction of the synthesis to studies with verified blinded outcome assessment; and (3) iterative leave-one-out (LOO) analysis.

#### 2.5.8. Clinical Translation Assessment

Clinical findings were extracted into a structured comparative matrix (mean, SD, median, *n*) and compared with preclinical effects. Because only two clinical RCTs provided complete continuous data, formal meta-analytic pooling was not performed; exploratory effect sizes (Hedges’ *g*) were calculated for individual trials to enable comparison with the preclinical findings.

#### 2.5.9. Publication Bias

To reduce artifactual mathematical correlation between standardized effect sizes and their standard errors, publication bias was evaluated using a modified Egger linear regression test with sample-size-based precision (1/√*N_total_*) as the predictor. Funnel plot asymmetry was assessed both visually and statistically for pools with *k* ≥ 10.

## 3. Results

### 3.1. Study Selection

The study selection process is presented in the PRISMA 2020 flow diagram (Figure 1). Following deduplication and sequential screening of titles/abstracts and full texts, 42 studies met the eligibility criteria and were included in the systematic review. These comprised 38 preclinical in vivo animal studies, 3 human clinical studies, and 1 veterinary randomized controlled trial (RCT) in client-owned dogs.

Of the 38 preclinical studies, 25 studies reported sufficient continuous data to permit quantitative comparison between intervention and control groups for time to hemostasis and/or blood loss and were therefore included in the meta-analysis. These 25 studies contributed 67 distinct intervention-versus-control pairwise comparisons, because several studies evaluated more than one intervention, control condition, outcome, or experimental subgroup. The remaining 13 preclinical studies did not provide complete or extractable continuous data suitable for quantitative pooling and were therefore included only in the qualitative synthesis.

The 3 human clinical studies and 1 veterinary RCT were not pooled with the preclinical animal studies and were analyzed separately using structured narrative and tabular synthesis, as described in Section 3.6.

### 3.2. Study Characteristics

The 42 included studies were published between 2017 and 2025, with the highest publication counts in 2023 (*n* = 12) and 2025 (*n* = 9), reflecting recent growth in research on visceral bioadhesives (Figure 2). Study designs comprised 3 human clinical studies, including two active-controlled RCTs (Bochicchio et al. [15] and Zhang et al. [35]) and one retrospective cohort (Fujikawa et al. [17]); 1 veterinary clinical RCT in dogs undergoing hepatic surgery [36]; and 38 preclinical in vivo animal studies, often accompanied by in vitro cytocompatibility and mechanical characterization.

Preclinical in vivo experiments were conducted predominantly in rats (*n* = 26, 62%), followed by pigs (*n* = 8, 19%), mice, rabbits, and dogs. Hepatic injury models included parenchymal punch-biopsy defects, standardized scalpel incisions, and partial wedge or lobar resections. The most common primary biopolymer matrices were chitosan (*n* = 14, 33%), gelatin (*n* = 7, 17%), fibrin (*n* = 5, 12%), collagen (*n* = 4, 10%), and alginate (*n* = 4, 10%); the remaining studies investigated oxidized cellulose composites, microporous polysaccharide spheres, and citrus pectin (Figure 3). Complete study characteristics are summarized in Table 2.

The evidence base was geographically concentrated: nearly half of all studies originated from China (*n* = 20, ~48%), followed by the United States (*n* = 9, ~21%), Israel (*n* = 3, ~7%), and Spain (*n* = 3, ~7%), with single studies from seven additional countries (Figure 4). Furthermore, 86% of included articles were published in 2021 or later. Data availability varied substantially across outcome domains (Figure 5): acute hemostatic efficacy and in vitro/in vivo biocompatibility were reported in >85% of studies, whereas quantitative mechanical adhesion and long-term biliary sealing were evaluated in fewer than 45% of publications.

### 3.3. Risk of Bias

Assessment of the 38 preclinical animal studies using the SYRCLE tool identified substantial methodological reporting limitations (Table 3). Allocation concealment and randomized housing were rated as unclear risk in all studies (*n* = 38). Blinding of caregivers/investigators during surgery was rated as high risk in 86.8% of studies (*n* = 33) because the adhesive materials were visually distinguishable from control gauze or hemostatic sponges. Blinding of outcome assessors was confirmed in only 21.1% of studies (*n* = 8), with the remaining 78.9% (*n* = 30) rated as unclear risk. Incomplete outcome data (attrition bias) and selective outcome reporting were judged to be at low risk across all 38 studies. For the clinical studies, key sources of bias included lack of blinded outcome assessment for subjective bleeding scores [36], absence of stratified randomization [15], and retrospective confounding [17]. Complete risk-of-bias appraisals were performed for all study types. For the 38 preclinical animal studies, domain-level SYRCLE judgments are summarized in Table 3, and the full study-by-domain matrix is provided in Supplementary Table S1. For human clinical evidence (Table 4), the two RCTs (Bochicchio et al. [15]; Zhang et al. [35]) were rated as having some concerns overall with RoB 2, driven primarily by open-label outcome measurement in the surgical setting, whereas other RoB 2 domains were generally low risk. The retrospective cohort (Fujikawa et al. [17]) was rated at moderate overall risk with ROBINS-I because of residual confounding and non-blinded outcome assessment. The veterinary RCT in client-owned dogs (Anderson et al. [36]) was appraised using RoB 2 adapted to veterinary trials and was judged to have a high risk of bias for outcome measurement owing to unblinded subjective bleeding scores, with low risk for the randomization process and missing outcome data.

### 3.4. Material Class Synthesis: Efficacy and Mechanical Properties

Table 5 summarizes pooled hemostatic effect estimates stratified by material class. For time to hemostasis versus negative control, the raw mean differences were MD = +688.3 s for protein-based systems (*k* = 7), MD = +178.7 s for polysaccharide-based systems (*k* = 3), and MD = +232.5 s for composite/modified systems (*k* = 4). Versus standard hemostats, the corresponding MDs were +15.3 s (protein-based, *k* = 6), +71.0 s (polysaccharide-based, *k* = 6), and +85.8 s (composite/modified, *k* = 5). For intraoperative blood loss versus negative control, the pooled RoMs were 11.50 for protein-based systems (*k* = 4), 4.12 for polysaccharide-based systems (*k* = 6), and 4.49 for composite/modified systems (*k* = 4); versus standard hemostats, the corresponding RoMs were 1.83 (protein-based, *k* = 7), 2.15 (polysaccharide-based, *k* = 7), and 2.09 (composite/modified, *k* = 6).

#### 3.4.1. Selected Individual Studies Illustrate This Variability

A carboxymethyl chitosan/gelatin/oxidized alginate hydrogel (CMC-GEL/OSA) reduced bleeding time by ~84% and blood loss by ~82% relative to untreated controls [38]. A TEMPO-oxidized cellulose nanofiber/collagen/chitosan aerogel (TCNF/COL/CS) and a peptide-polysaccharide hydrogel (CP100) showed greater hemostatic efficacy than the commercial products Surgicel and Surgiflo [12,30]. However, not all comparisons favored investigational bioadhesives: in an American Association for the Surgery of Trauma (AAST) grade V liver injury model, microporous polysaccharide spheres (PerClot) stabilized clot adhesion during the subacute period more effectively than Hemopatch [4], and GelMAC produced slower hemostasis than Surgicel in one incision model [9].

#### 3.4.2. Adhesive and Cohesive Mechanical Strength

Quantitative mechanical strength was evaluated in 18 studies; test methods (lap shear, tensile pull-off, peel, and burst pressure) and units (kPa, MPa, mmHg, J·m^−2^) varied substantially, precluding quantitative pooling (Table 6). Across 18 measurements from 11 studies reporting shear adhesive strength in kPa, values ranged from 0.66 to 104.9 kPa. Protein-based hydrogels had the highest median shear strength (~42.0 kPa), with the largest reported values for a collagen-tannic acid hydrogel (CT–5, 104.9 kPa under wet conditions) [23] and a batroxobin-loaded gelatin hydrogel (HAD, 93.5 kPa) [10]. Polysaccharide and composite systems had lower median values (10.6 and 17.2 kPa, respectively), with the lowest value reported for an ibuprofen-conjugated chitosan matrix (0.66 kPa) [45].

#### 3.4.3. Burst Pressure

Burst pressure was quantified in 14 studies. Several formulations withstood pressures substantially above physiological portal (8–12 mmHg) and arterial (100–120 mmHg) pressures, including Carbopol/chitosan microneedle arrays (~480 mmHg) [28], CP100 hydrogel (~328 mmHg) [12], and a pectin tissue patch (~234 mmHg) [46]. Chemical functionalization enhanced wet-tissue adhesion in several systems, including catechol-functionalized GelMAC [9], whereas macroporosity promoted rapid hemostasis through fluid uptake and cellular hemoconcentration [31,44,47]. The wide variation in reported metrics and units (e.g., interfacial fracture toughness of 473.7 J·m^−2^ for adhesive cryogel particles [32] and tensile modulus of 15.2 MPa for cellulose composites [6]) highlights the lack of standardized biomechanical testing protocols for visceral sealants.

### 3.5. Biocompatibility: Narrative Synthesis

Biocompatibility was evaluated in 36 of the 42 included studies (86%), and the studies generally reported favorable tissue tolerance. In vitro cytocompatibility assays using L929 fibroblasts, NIH/3T3 cells, human umbilical vein endothelial cells (HUVECs), and primary hepatocytes reported cell viability generally above 80%, with many values in the 80–95% range; in vitro hemolysis remained below the 5% threshold (typically 0.12–2.0%). In vivo histological assessments showed mild-to-moderate acute inflammatory infiltration that resolved over 2–4 weeks, with physiological enzymatic or hydrolytic biodegradation completed within 21–56 days [32,39,40]. Several biomaterial platforms also promoted macrophage polarization toward an anti-inflammatory M2 phenotype, supported hepatic parenchymal regeneration, or reduced postoperative adhesion formation through biofunctional additives such as stem cell secretome, mesenchymal stem cell apoptotic vesicles, and ibuprofen [11,27,45]; others exhibited intrinsic broad-spectrum antimicrobial activity [12,21,30].

### 3.6. Clinical Studies and Preclinical Comparison

Four clinical or veterinary investigations were identified: three human clinical studies (two RCTs and one retrospective cohort) and one veterinary RCT; their outcomes are summarized in Table 7. In a phase 1/2 human RCT, the investigational biodegradable fibrin sealant film sFilm-FS showed hemostatic efficacy comparable to that of the active control TachoSil [15]. In another RCT, oxidized non-regenerated cellulose gauze was non-inferior to oxidized regenerated cellulose in patients undergoing hepatectomy [35]. In a retrospective cohort of robotic hepatectomy, microporous polysaccharide hemospheres showed a favorable safety profile and procedural efficiency [17]. In the veterinary RCT, an adhesive gelatin sponge performed significantly better than a plain collagen sponge with respect to time to hemostasis and patch retention [36].

For the two clinical RCTs reporting complete continuous outcome data, exploratory standardized mean differences (Hedges’ *g*) were calculated. For time to hemostasis, the clinical effect size was *g* = +0.42 (SE = 0.23; *n* = 37 vs. 38) [35]; for intraoperative blood loss, it was *g* = −0.41 (SE = 0.34; *n* = 17 vs. 16) [15]. Neither effect was statistically significant because the 95% CIs crossed zero, and both were substantially smaller than the pooled preclinical animal effect sizes (*g* ≈ +2.8 to +3.3). This discrepancy indicates a substantial translational gap: the marked efficacy observed in healthy animal models was not reproduced in the available human surgical studies. Formal meta-analysis of clinical data was precluded by the small number of trials and heterogeneous outcome measures; these estimates should therefore be interpreted as exploratory.

### 3.7. Meta-Analysis (Preclinical Data): Overall Results

The quantitative meta-analysis included 25 preclinical studies with complete continuous data, yielding 67 pairwise comparisons. Random-effects models were fitted using REML variance estimation and HKSJ confidence intervals. Pooled estimates for the four primary analytical pools are summarized in Table 8 and shown in forest plots stratified by material class (Figure 6, Figure 7, Figure 8 and Figure 9). Raw mean difference (MD, seconds) was used for time to hemostasis, whereas ratio of means (RoM) was used for blood loss and time. The REML + HKSJ approach provided conservative confidence intervals that account for substantial between-study heterogeneity.

The absolute time to hemostasis in the included preclinical experiments ranged from approximately 2 to 582 s (median ~41 s), spanning nearly three orders of magnitude. This wide range illustrates substantial clinical and methodological diversity and is consistent with the high *I*^2^ values.

#### 3.7.1. Time to Hemostasis

Compared with untreated negative controls (no treatment/physiological saline), bioadhesives significantly shortened time to hemostasis (MD = +419.53 s, 95% CI: 164.44 to 674.62 s; *k* = 14; *p* = 0.0035; RoM = 5.07, 95% CI: 3.10 to 8.29), with pronounced statistical heterogeneity (*I*^2^ = 99%). Compared with standard active hemostats (Surgicel, TachoSil, Arista, etc.), the pooled effect remained statistically significant in preclinical models (MD = +56.05 s, 95% CI: 20.14 to 91.96 s; *k* = 17; *p* = 0.0044; RoM = 1.91, 95% CI: 1.31 to 2.79; *I*^2^ = 99%). Sensitivity analysis using standardized mean differences showed effects in the same direction (Hedges’ *g* = +9.72 vs. negative control and *g* = +3.30 vs. active standard).

#### 3.7.2. Blood Loss

Bioadhesive application significantly reduced intraoperative blood loss. Compared with untreated negative controls, blood loss was reduced 5.68-fold (RoM = 5.68, 95% CI: 3.79 to 8.51; *k* = 14; *p* < 0.0001; *I*^2^ = 96%); compared with standard active hemostats, it was reduced 2.05-fold (RoM = 2.05, 95% CI: 1.48 to 2.86; *k* = 20; *p* = 0.0002; *I*^2^ = 96%). Sensitivity analyses using standardized mean differences yielded Hedges’ *g* = +5.37 and +2.75, respectively. Wide 95% prediction intervals indicate substantial variability across liver injury models.

### 3.8. Subgroup Analysis by Material Class

Random-effects meta-regression (Knapp-Hartung F-test) showed no statistically significant differences in hemostatic efficacy among the three material classes (protein-based, polysaccharide-based, and composite/modified) for either time to hemostasis (*p* = 0.178 vs. negative control; *p* = 0.213 vs. standard hemostat) or blood loss (*p* = 0.064 vs. negative control; *p* = 0.852 vs. standard hemostat) (Table 9). Although polysaccharide matrices showed numerically larger effects in some analyses (e.g., RoM = 2.15 vs. active hemostats), no statistically significant between-class differences were detected. Between-study heterogeneity nevertheless remained substantial across material classes, including *I*^2^ = 94% for protein-based and 95% for polysaccharide-based systems in the blood-loss comparison versus negative control.

Within material subgroups, statistical heterogeneity generally remained high to extreme (*I*^2^ up to 96–99%), likely reflecting differences in defect geometry, formulation and delivery, application technique, bleeding severity, and outcome-assessment protocols.

### 3.9. Subgroup Analysis by Hemostatic Mechanism

The 25 preclinical interventions were distributed across four primary hemostatic mechanisms (Table 10): coagulation activation (*n* = 3), chemical adhesion (*n* = 8), physical absorption (*n* = 6), and combined mechanisms (*n* = 8). Subgroup pooling using REML + HKSJ models (Table 11) and random-effects meta-regression showed no statistically significant differences among mechanisms for time to hemostasis versus standard hemostat (*p* = 0.082) or blood loss versus standard hemostat (*p* = 0.090). All four mechanisms produced hemostatic effects; however, no individual mechanism showed statistically significant superiority over active standard hemostats, and observed differences should therefore be considered hypothesis-generating.

### 3.10. Sources of Heterogeneity: Meta-Regression

Because substantial heterogeneity persisted within material classes and hemostatic mechanisms, potential moderators of between-study variance were examined using mixed-effects meta-regression with animal species (rodents vs. large animals) and injury geometry (resection vs. localized defect) as covariates (Table 12). Neither covariate explained a statistically significant proportion of between-study variance (all *Q_M_ p* > 0.10; *R*^2^ ≈ 0%), indicating that species and gross injury geometry alone did not account for the observed variation in effect estimates. Residual heterogeneity likely reflects a multifactorial combination of formulation microarchitecture, application pressure and volume, operator technique, and bleeding severity.

### 3.11. Sensitivity Analysis by Risk of Bias

The stability of the pooled findings was assessed using three predefined sensitivity analyses (Table 13). First, excluding studies at high risk of bias in key SYRCLE domains produced similar effect estimates (e.g., for time to hemostasis vs. standard hemostats, Hedges’ *g* changed from +2.59 [*k* = 17] to +2.84 [95% CI: 0.10 to 5.59; *k* = 16]), suggesting that overall statistical significance was not driven by the excluded high-risk studies. Second, restricting the synthesis to studies with verified blinded outcome assessment (Deineka et al. [40], Eskildsen et al. [16], Guo et al. [42]) reduced the pooled effect for time to hemostasis versus standard hemostats to Hedges’ *g* = +1.29 (95% CI: −6.31 to +8.90; *t* = 0.73; *p* = 0.541; *k* = 3), with the CI crossing the null. Similarly, for blood loss versus standard hemostats, the blinded subset (Eskildsen et al. [16], Deineka et al. [40]) yielded *g* = +4.43 (95% CI: −10.50 to +19.37; *p* = 0.283; *k* = 2). These findings suggest that lack of observer blinding may introduce detection bias; however, because only 2–3 studies used verified blinding, the subset estimates are highly imprecise. Third, iterative leave-one-out analyses showed that no individual study exerted undue influence on the pooled estimates (all *p* < 0.01).

### 3.12. Publication Bias Assessment

A modified Egger regression test using sample-size-based precision (1/√*N_total_*) did not detect statistically significant funnel plot asymmetry in any of the four primary analytical pools (*p* = 0.071 to 0.989; Table 14). Publication bias was therefore not confirmed statistically; however, it cannot be excluded given the visual funnel-plot asymmetry (Figure 10, Figure 11, Figure 12 and Figure 13), small study sample sizes, geographic concentration of the evidence base (nearly 70% from China and the USA), and the predominance of positive findings in the materials-science literature.

### 3.13. Certainty of Evidence (GRADE)

According to the adapted preclinical animal GRADE framework (Table 15), all four primary outcomes were downgraded for serious risk of performance/detection bias (−1; lack of blinding in up to 79–87% of studies and unclear allocation concealment in 100%), serious inconsistency (−1; extreme heterogeneity, *I*^2^ = 96–99%, and 95% prediction intervals that included the null value, including −90.37 to +202.47 s for time to hemostasis and 0.48 to 8.74 for the blood-loss RoM versus standard hemostats), and serious indirectness (−1; young, healthy rodent or porcine liver tissue with standardized injuries versus diseased human livers with cirrhosis or post-chemotherapy friability). Consequently, the overall certainty of evidence was rated as very low for time to hemostasis and blood loss versus standard hemostats and as low to very low for comparisons with negative controls.

## 4. Discussion

### 4.1. Principal Findings and Clinical Interpretation

A clear distinction is required between statistical effects observed in controlled experimental models and proven clinical utility in surgical practice. In preclinical models, bio-based adhesives and sealants significantly shortened time to complete hemostasis on liver parenchyma by an average of ~420 s relative to untreated controls and by ~56 s relative to standard active hemostats (oxidized cellulose, collagen sponges, fibrin sealants), while reducing blood loss by 5.7-fold and 2.05-fold, respectively. However, given the methodological limitations of the primary studies and the low certainty of evidence according to GRADE, these findings should be described as promising preclinical efficacy rather than established clinical superiority.

Surgical procedures involving the liver, including anatomical and non-anatomical resections, tumor enucleation, living-donor transplantation, and repair of traumatic lacerations, traverse highly vascularized parenchyma containing fragile biliary radicles and sinusoids with limited intrinsic contractility. Conventional techniques such as parenchymal suturing, clip ligation, and monopolar or bipolar electrocautery are limited by tissue friability, the risk that sutures will cut through the parenchyma, and secondary ischemic injury. Under these conditions, bioadhesives may provide a useful adjunct by forming an elastic, biocompatible barrier that conforms to the transection surface and seals microvascular and biliary openings without compressing the parenchyma. Nevertheless, the present findings show considerable heterogeneity across polymer formulations and hemostatic mechanisms.

### 4.2. Material Class Specificity and Hemostatic Mechanisms

Stratification by chemical composition showed substantial preclinical hemostatic activity across all three material classes (protein-based, polysaccharide-based, and composite/modified). Protein-based systems (fibrin, collagen, gelatin) showed the highest in vitro lap-shear adhesive strength (median ~42 kPa; maximum reported values 93.5–104.9 kPa), although they did not consistently outperform other systems in models of severe parenchymal hemorrhage. Polysaccharide matrices (chitosan, alginate, cellulose) showed numerically larger effects in some analyses (e.g., blood-loss RoM = 2.15 vs. active hemostats), but meta-regression detected no statistically significant between-class differences (*p* = 0.064 and *p* = 0.852 for blood loss; *p* = 0.178 and *p* = 0.213 for time to hemostasis). Between-study heterogeneity also remained substantial within both protein-based and polysaccharide-based groups (*I*^2^ = 94% and 95%, respectively, for blood loss vs. negative control). Composite and chemically modified formulations also reduced blood loss (RoM = 4.49 vs. control; RoM = 2.09 vs. standard) but showed considerable dispersion (*I*^2^ = 74–96%), suggesting that performance may depend strongly on crosslinking density, curing kinetics, and operator handling.

Stratification by functional hemostatic mechanism suggested that physical absorption and chemical tissue adhesion were associated with relatively consistent reductions in blood loss. Formulations relying solely on coagulation activation (thrombin/fibrin preparations) did not show superiority over active commercial hemostats (RoM = 0.86, 95% CI: 0.10 to 7.03). These findings suggest that, in sharp parenchymal liver transections, rapid physical localization and tamponade of blood flow may be at least as important as enzymatic amplification of the coagulation cascade.

### 4.3. The Translational Gap: Preclinical Efficacy Versus Clinical Reality

A major finding of this systematic review is the marked divergence between preclinical animal outcomes and clinical trial results, consistent with a substantial translational gap. Pooled preclinical effects were large (Hedges’ *g* ≈ +2.8 to +3.3), whereas exploratory effect sizes from the available human RCTs were small and not statistically significant: *g* = +0.42 (95% CI: −0.03 to +0.87) for time to hemostasis [35] and *g* = −0.41 (95% CI: −1.08 to +0.26) for blood loss [15]. None of the included clinical trials demonstrated definitive superiority of novel bioadhesives over contemporary active surgical standards.

This translational discrepancy may reflect a combination of biological, operative, and methodological factors. Experimental studies in rodents or healthy swine generally use young, uncompromised liver tissue with standardized incisions or punch defects. By contrast, major liver resections in clinical oncology and hepatology are often performed in older patients with comorbidities and underlying steatosis, fibrosis or cirrhosis, or sinusoidal obstruction syndrome after neoadjuvant systemic chemotherapy. These conditions may increase tissue friability and impair coagulation. Moreover, real-world surgical hemostasis is inherently multimodal and may involve temporary vascular occlusion (Pringle maneuver), ultrasonic dissection, bipolar coagulation, and vascular clip ligation. In this setting, the incremental contribution of a topical bioadhesive may be smaller than in isolated experimental models. Methodologically, preclinical animal studies are limited by substantial reporting deficiencies, frequent absence of blinding, and possible publication bias, all of which may inflate effect estimates.

### 4.4. Adhesive Strength, Biocompatibility, and the Biliary Sealing Gap

Comparison of in vitro biomechanical metrics with in vivo hemostatic performance indicates that static tensile or lap-shear adhesive strength is an imperfect surrogate for clinical performance. Protein hydrogels with high in vitro shear strength (>90–100 kPa) did not show proportionally faster in vivo hemostasis than porous polysaccharide sponges with more modest static adhesion. In an actively bleeding surgical field, the rate of cohesive network formation under wet conditions and blood pressure may be more clinically relevant than the ultimate dry tensile strength of a fully crosslinked polymer.

The overall biocompatibility profile of the investigated bio-based adhesives was generally favorable: natural biopolymers underwent gradual physiological biodegradation within 21–56 days without reported chronic granulomatous foreign-body reactions. Nonetheless, chemical crosslinking reagents such as glutaraldehyde and polyisocyanates, as well as non-targeted photoinitiators, may cause focal hepatocyte necrosis. An important gap in the preclinical literature is the systematic evaluation of bile-duct sealing. Postoperative bile leakage (including bilioma and biliary peritonitis) represents the second most frequent organ-specific complication after hepatectomy; however, most animal studies focused almost exclusively on vascular hemostasis, leaving biliary sealing efficacy largely unverified.

### 4.5. Methodological Quality and Risk of Bias

Methodological limitations in the preclinical literature may contribute substantially to effect inflation. SYRCLE assessment showed that allocation concealment was unclear in all studies and that outcome-assessor blinding was confirmed in only 21.1%. Because the primary endpoint—visually determined time to complete hemostasis—is inherently subjective, lack of blinding may introduce important detection bias.

The sensitivity analysis was consistent with this concern. Restricting the synthesis to studies with verified blinded outcome assessment (Deineka et al. [40], Eskildsen et al. [16], Guo et al. [42]) reduced the pooled effect for time to hemostasis versus standard hemostats from a baseline Hedges’ *g* of +2.59 (95% CI: 0.07 to 5.10) to *g* = +1.29 (95% CI: −6.31 to +8.90; *p* = 0.541), with the CI crossing the null. Additional potential sources of bias include experimental pseudoreplication, in which multiple liver lesions in one animal are analyzed as independent observations, and very small group sizes (*n* = 3–6), which can produce unstable variance estimates. REML estimation and HKSJ adjustments were therefore used to provide more conservative inference under substantial heterogeneity and small study numbers.

### 4.6. Clinical Safety, Non-Hemorrhagic Outcomes, and Ischemia–Reperfusion Organoprotection

In clinical liver surgery, morbidity is determined not only by acute blood loss but also by non-hemorrhagic complications such as bile leakage, perihepatic abscesses, and relaparotomy. In the included clinical cohorts, microporous polysaccharide spheres and oxidized cellulose showed favorable safety profiles without severe (Clavien–Dindo > II) complications [17,35], whereas studies of fibrin sealants reported occasional bile leaks and bowel obstruction requiring reintervention [17]. Postoperative abdominal drainage also varied across materials and commercial formulations: in human trials, cellulose gauze was associated with median drainage volumes of 68–75 mL on postoperative day 1 [35], whereas fibrin sealant patches showed greater variability, from 97.0 ± 61.1 mL for TachoSil to 522.7 ± 532.5 mL for sFilm-FS [15].

Parenchymal hemostasis is particularly important during major hepatectomy, in which blood-loss reduction often relies on intermittent hepatic inflow occlusion using the Pringle maneuver. Inflow occlusion induces warm hepatic ischemia, and subsequent reperfusion can trigger ischemia–reperfusion injury (IRI). In this setting, topical bioadhesives could serve as adjuncts by accelerating parenchymal sealing and thereby reducing the cumulative duration or number of Pringle-clamping cycles, potentially limiting ischemic stress on the future liver remnant. Atraumatic sealing may also reduce the need for aggressive electrocoagulation and parenchymal transfixion, limiting mechanical injury and release of damage-associated molecular patterns (DAMPs) that contribute to post-reperfusion inflammation. Although pathophysiologically plausible, this hepatoprotective hypothesis remains unverified because ischemia duration, Pringle cycles, and hepatic transaminase kinetics (alanine aminotransferase [ALT] and aspartate aminotransferase [AST]) were not systematically reported in the available literature.

### 4.7. Future Perspectives and Recommendations for Clinical Research

Bridging the translational gap will require substantial improvements in study design. Preclinical investigations should adhere closely to Animal Research: Reporting of In Vivo Experiments (ARRIVE 2.0) and SYRCLE guidance, including prospective sample-size calculations, formal randomization, verified allocation concealment, and standardized video recording with blinded outcome assessment. Studies should prioritize clinically relevant large-animal models, such as porcine hepatectomy, with induced coagulopathy and formal bile-duct pressure-leak testing. Preclinical studies should also record vascular occlusion duration, the number of Pringle-maneuver cycles, ALT/AST dynamics, and inflammatory markers to investigate potential mitigation of IRI. Clinical validation requires adequately powered multicenter RCTs with active comparators (TachoSil, Surgicel), standardized bleeding-severity scales (Validation of Intraoperative Bleeding [VIBe] or Surface Bleeding Severity Scale [SPOT GRADE]), and rigorous long-term monitoring of bile leakage, bilioma formation, drain volume, transfusion requirements, and 30-day morbidity. As an illustrative sample-size benchmark for a continuous primary endpoint (e.g., time to hemostasis or intraoperative blood loss), detection of a small-to-moderate standardized effect (Cohen’s d ≈ 0.35–0.40)—consistent with the exploratory clinical estimates observed in the available RCTs (Hedges’ *g* ≈ 0.4) and substantially smaller than pooled preclinical effects—at two-sided α = 0.05 and 80–90% power yields approximately 100–170 participants per arm under a two-sample t-test framework (*n* per arm ≈ 2[(zα/2 + zβ)/d]^2^). Accordingly, we recommend planning future trials with at least *n* ≥ 100–150 patients per arm, with final numbers confirmed by a protocol-specific power calculation that incorporates the primary endpoint, expected variance, clinically meaningful difference, attrition, and multicenter design effects.

### 4.8. Limitations of the Review and Evidence Base

This systematic review and its underlying evidence base have several limitations. Limitations of the primary literature include the predominance of small-animal studies with substantial reporting deficiencies and frequent lack of blinded outcome assessment; extreme statistical heterogeneity (*I*^2^ = 96–99%) arising from differences in injury models and dosing regimens; wide 95% prediction intervals that include the null value; and a scarcity of clinical RCTs (*k* = 3). Although the modified Egger regression did not detect statistically significant funnel plot asymmetry (*p* = 0.071–0.989), publication bias cannot be excluded. Review-level limitations include restriction of the search to open or publicly accessible resources (PubMed, Dimensions, Consensus, SciSpace) without institutional access to closed commercial databases (Embase, Scopus, Web of Science), retrospective reconstruction of initial identification counts, and use of common effect metrics (MD and RoM) to harmonize heterogeneous reporting scales. Under the adapted animal GRADE framework, the certainty of evidence was rated as low to very low across outcomes, supporting a cautious clinical interpretation.

## 5. Conclusions

In preclinical models of liver surgery, biologically derived adhesives, sealants, and topical hemostats significantly shortened time to hemostasis (MD = +419.5 s vs. untreated control; MD = +56.1 s vs. active hemostatic standard) and reduced intraoperative blood loss (RoM = 5.68 vs. control; RoM = 2.05 vs. standard). However, extreme between-study heterogeneity (*I*^2^ = 96–99%) and 95% prediction intervals that include the null value indicate substantial uncertainty about the magnitude of effect expected in an individual future surgical setting.

A pronounced translational gap remains: the large effect sizes observed in healthy animal models have not been reproduced in human surgical trials, in which clinical effect sizes were small and not statistically significant. Together with the frequent absence of blinded outcome assessment and the high risk of performance bias, this divergence supports interpreting preclinical findings as hypothesis-generating rather than as evidence of established clinical efficacy.

According to the adapted animal GRADE framework, the overall certainty of evidence was low to very low across the primary outcomes. All three material classes (protein-based, polysaccharide-based, and composite/modified systems) demonstrated substantial preclinical hemostatic effects; although polysaccharide and composite platforms showed numerically favorable results in some comparisons, meta-regression detected no statistically significant between-class superiority.

Topical bioadhesives may serve as useful adjuncts for perioperative organ protection during liver resection by potentially reducing the duration and number of Pringle-maneuver cycles and thereby limiting hepatic ischemia–reperfusion injury; however, this role requires targeted experimental validation. Routine clinical adoption will require adequately powered multicenter RCTs (illustratively *n* ≥ 100–150 per arm for detecting a small-to-moderate effect [Cohen’s d ≈ 0.35–0.40] at 80–90% power; final *n* should follow a protocol-specific calculation) with active comparators, standardized bleeding scales, and rigorous long-term monitoring of biliary leakage and infectious complications. Future preclinical research should also adhere closely to ARRIVE 2.0 and SYRCLE standards.

## Figures and Tables

**Figure 1:**
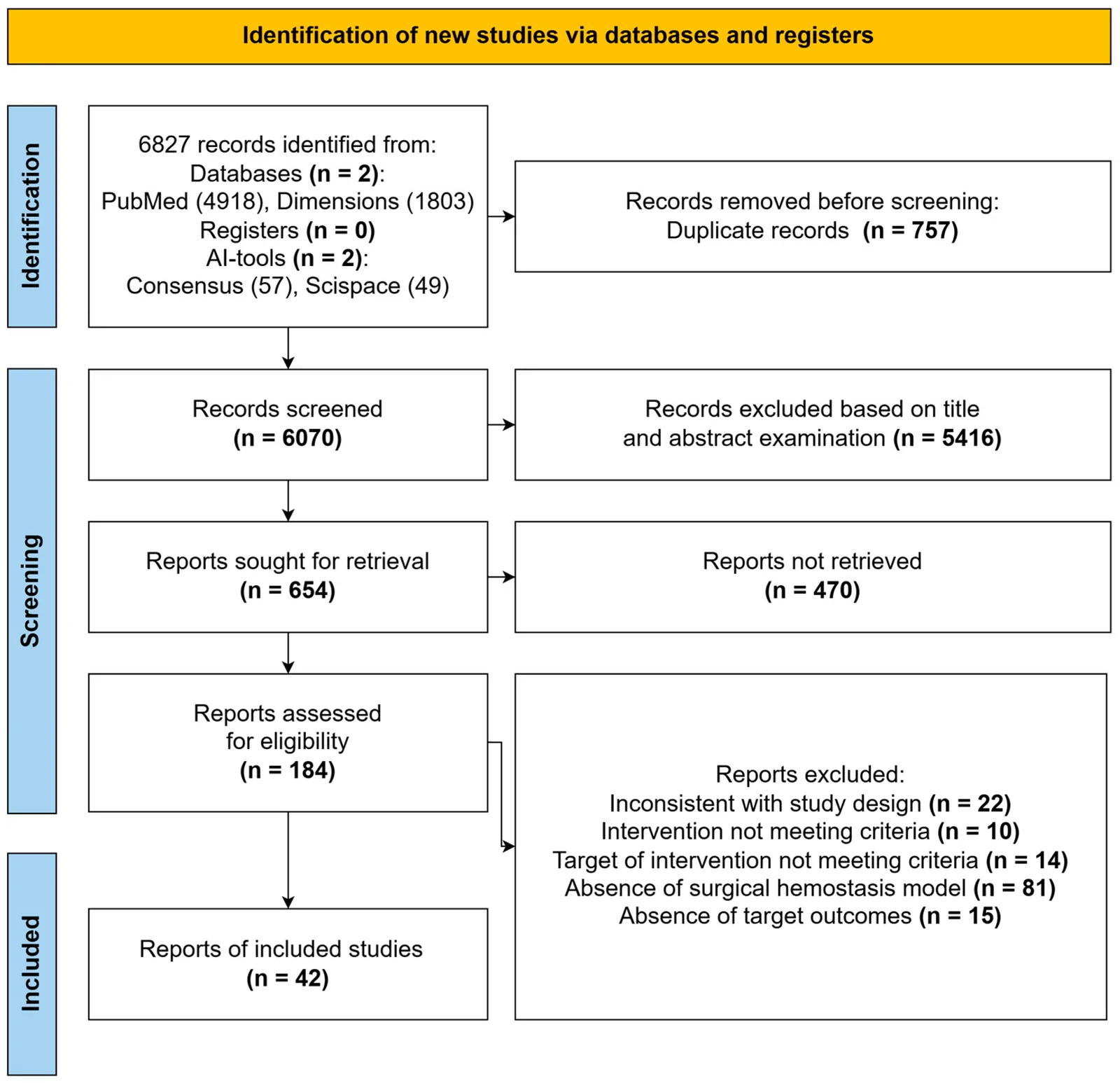
PRISMA 2020 flow diagram of study identification, screening, and inclusion. The diagram shows the sequential screening process leading to inclusion of 42 studies in the systematic review: 25 preclinical in vivo animal studies with continuous quantitative data eligible for meta-analysis (67 comparisons) and 4 clinical/veterinary studies synthesized narratively. Abbreviations: AI, artificial intelligence; PRISMA, Preferred Reporting Items for Systematic Reviews and Meta-Analyses; RCT, randomized controlled trial.

**Figure 2:**
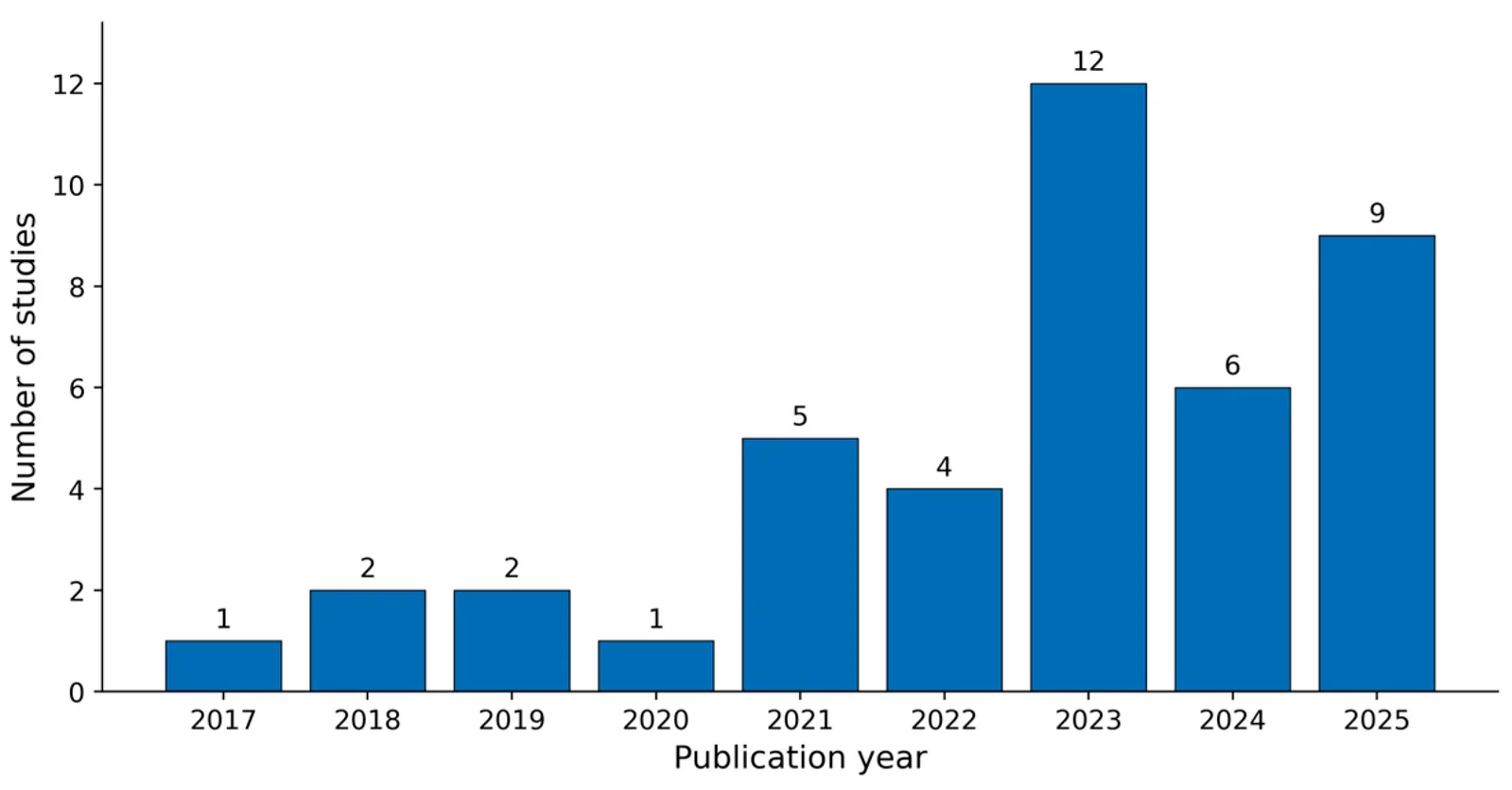
Distribution of included studies by publication year (2017–2025, *n* = 42). Publication activity increased markedly over time, with 86% of included studies published in 2021 or later. Abbreviation: *n*, number of studies.

**Figure 3:**
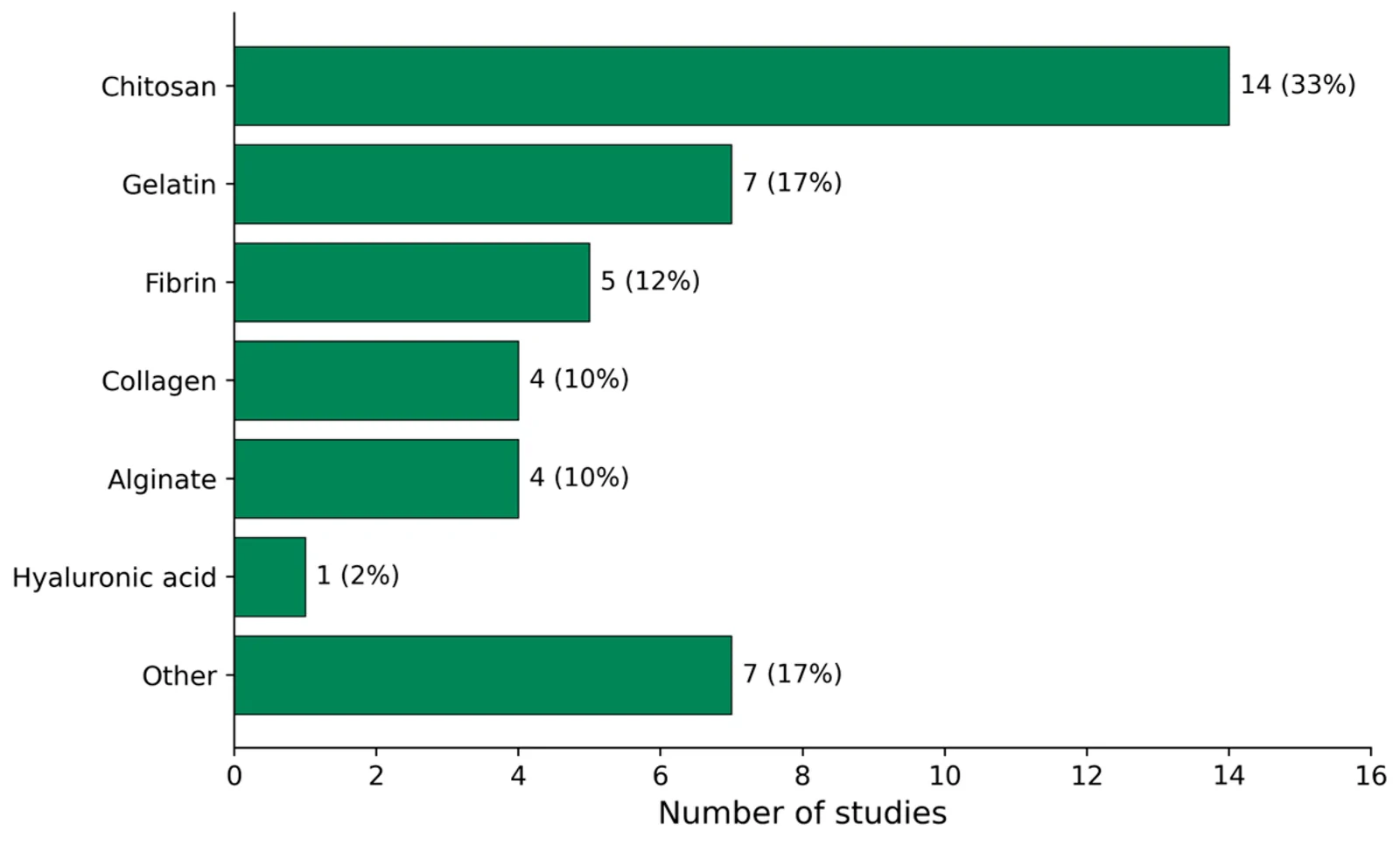
Distribution of included studies by primary biopolymer component (*n* = 42). Chitosan-based formulations (*n* = 14, 33%) were most common, followed by gelatin (*n* = 7, 17%), fibrin (*n* = 5, 12%), and collagen (*n* = 4, 10%). Abbreviation: *n*, number of studies.

**Figure 4:**
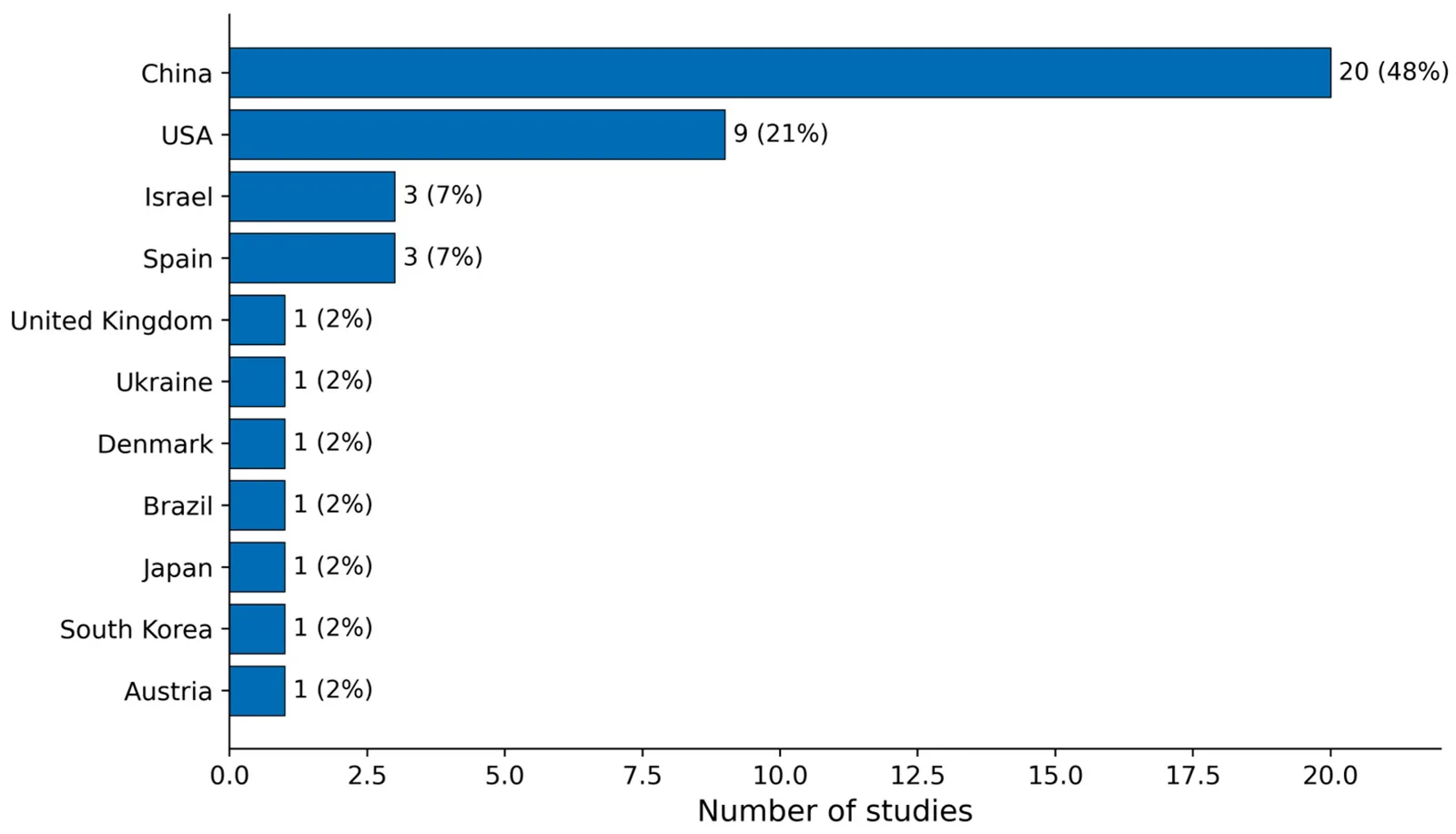
Geographic distribution of included studies according to the first author’s country of affiliation (*n* = 42). China (*n* = 20, ~48%) and the United States (*n* = 9, ~21%) together accounted for nearly 70% of the evidence base. Abbreviation: *n*, number of studies.

**Figure 5:**
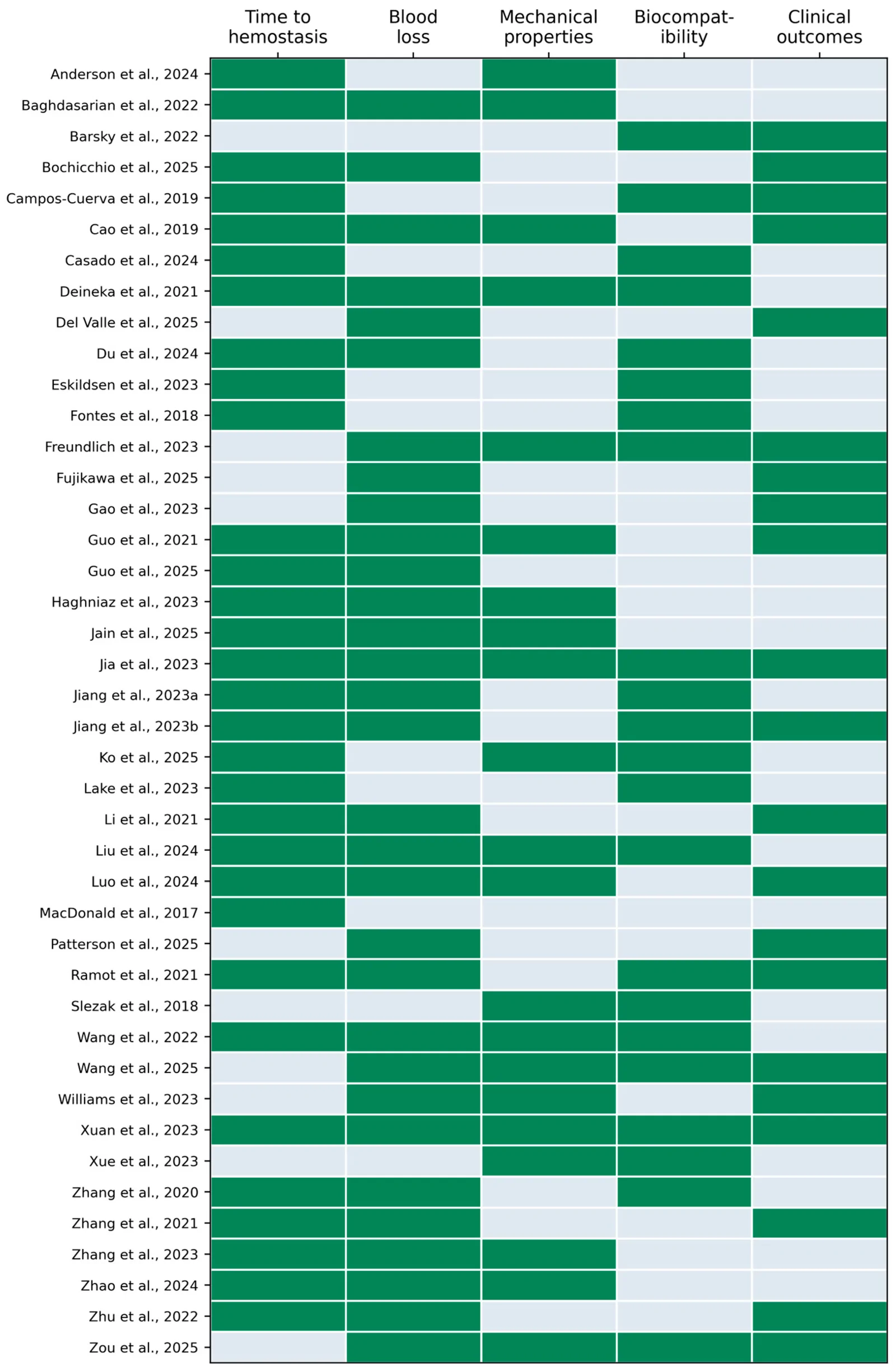
Data-availability heatmap across included studies (*n* = 42). Green shading indicates reported quantitative or qualitative data for the respective outcome domain; grey shading indicates no reported data. Acute hemostatic outcomes were widely reported, whereas mechanical and clinical outcome assessments were less consistently available [1,2,3,4,5,6,9,10,11,12,15,16,17,18,19,20,21,22,23,24,25,26,27,28,29,30,31,32,35,36,37,38,39,40,41,42,43,44,45,46,47,48].

**Figure 6:**
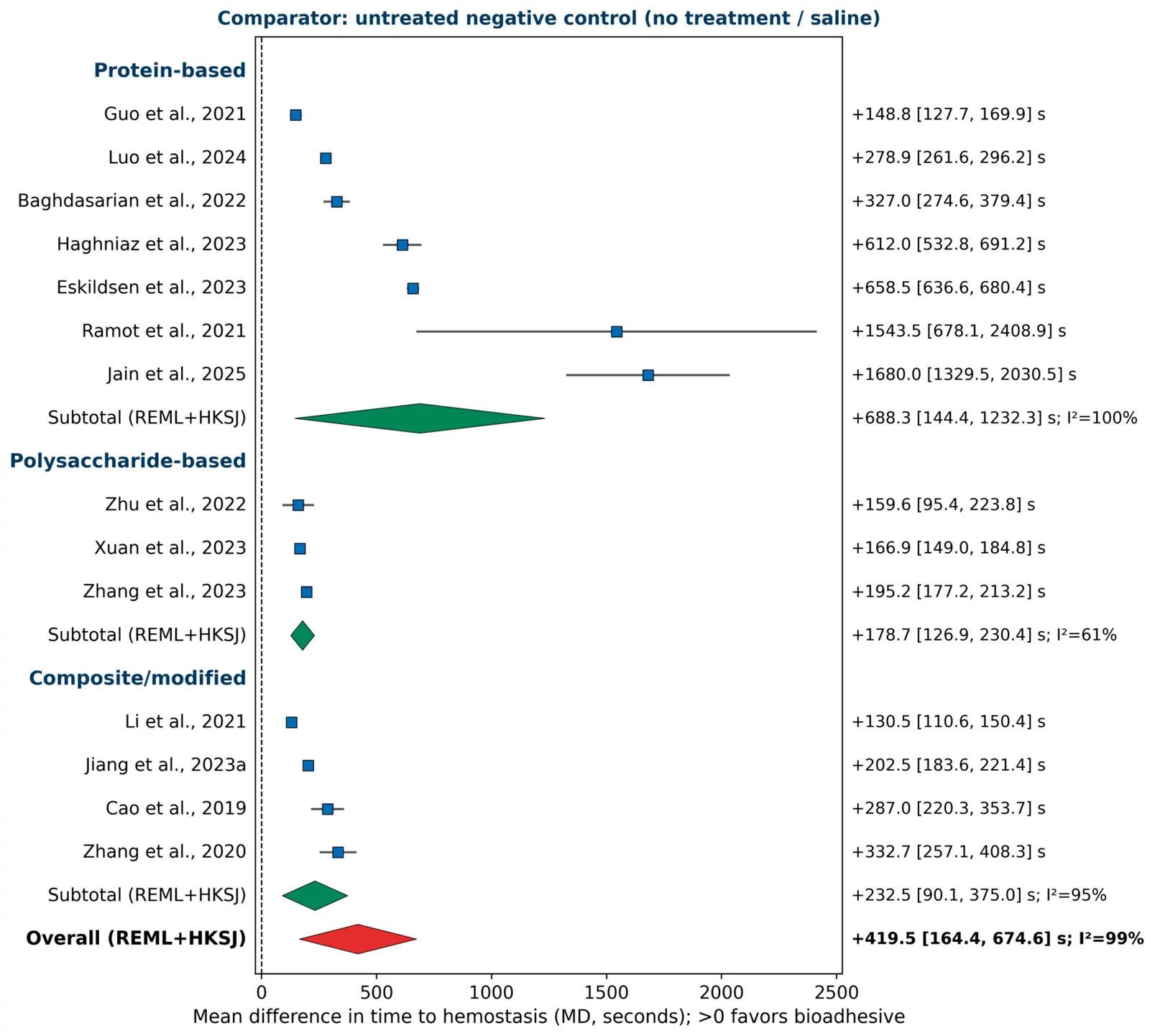
Forest plot of time to hemostasis (mean difference in seconds) stratified by material class: bioadhesive versus untreated negative control. Random-effects meta-analysis (REML + HKSJ): overall MD = +419.5 s (95% CI: 164.4 to 674.6 s); *I*^2^ = 99%, *τ*^2^ = 24,718.3; subgroup meta-regression *p* = 0.178. Green diamonds represent subgroup pooled estimates; the red diamond represents the overall pooled estimate. Horizontal error bars indicate 95% confidence intervals. Abbreviations: CI, confidence interval; HKSJ, Hartung–Knapp–Sidik–Jonkman; MD, raw mean difference; REML, restricted maximum likelihood; s, seconds [6,9,10,11,12,16,18,21,22,29,31,38,43,47].

**Figure 7:**
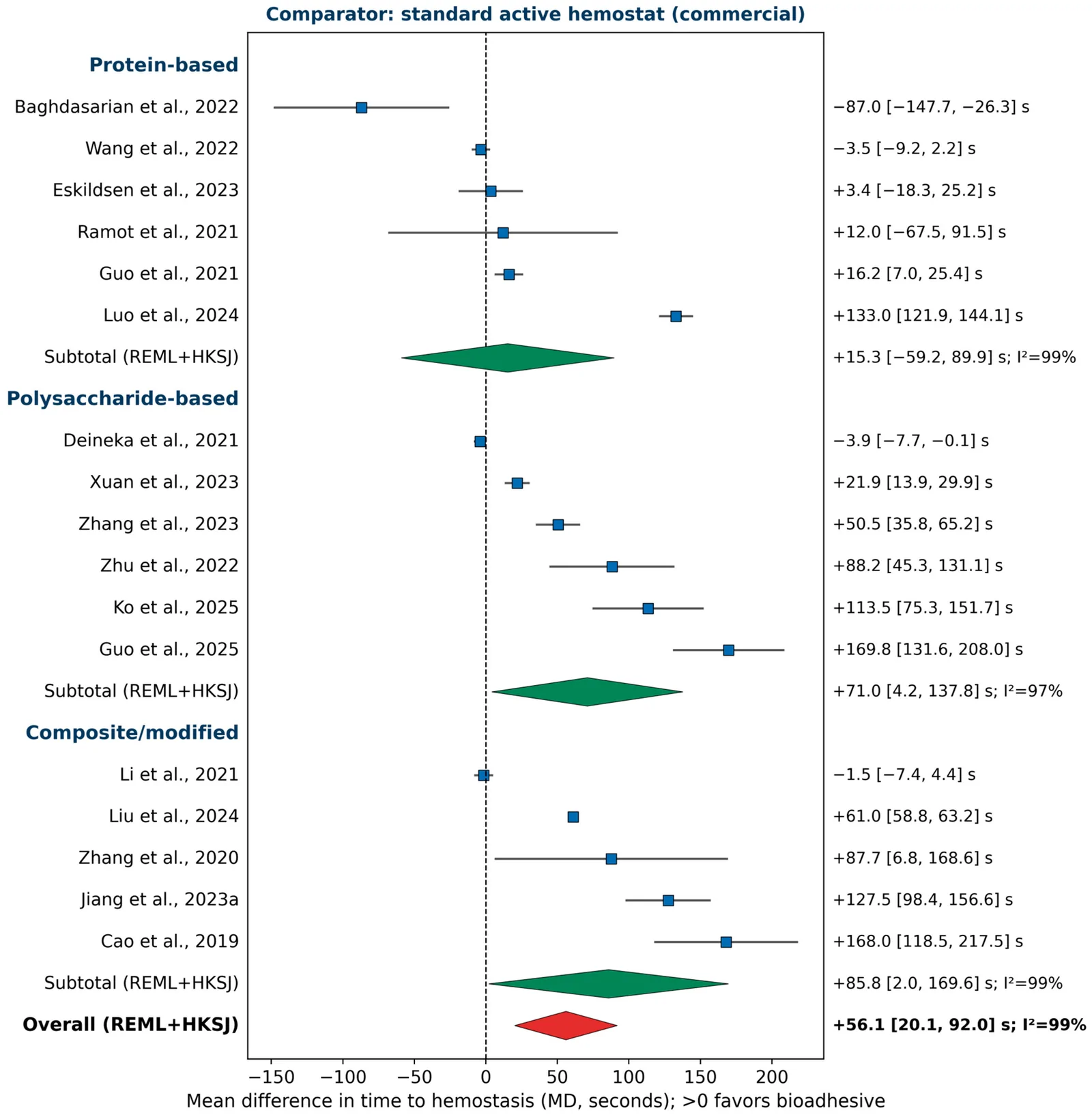
Forest plot of time to hemostasis (mean difference in seconds) stratified by material class: bioadhesive versus standard active hemostat. Random-effects meta-analysis (REML + HKSJ): overall MD = +56.05 s (95% CI: 20.14 to 91.96 s); *I*^2^ = 99%, *τ*^2^ = 2772.3; subgroup meta-regression *p* = 0.213. Green diamonds represent subgroup pooled estimates; the red diamond represents the overall pooled estimate. Abbreviations: CI, confidence interval; HKSJ, Hartung–Knapp–Sidik–Jonkman; MD, raw mean difference; REML, restricted maximum likelihood; s, seconds [6,9,10,11,12,16,18,22,25,30,31,38,40,42,43,44,47].

**Figure 8:**
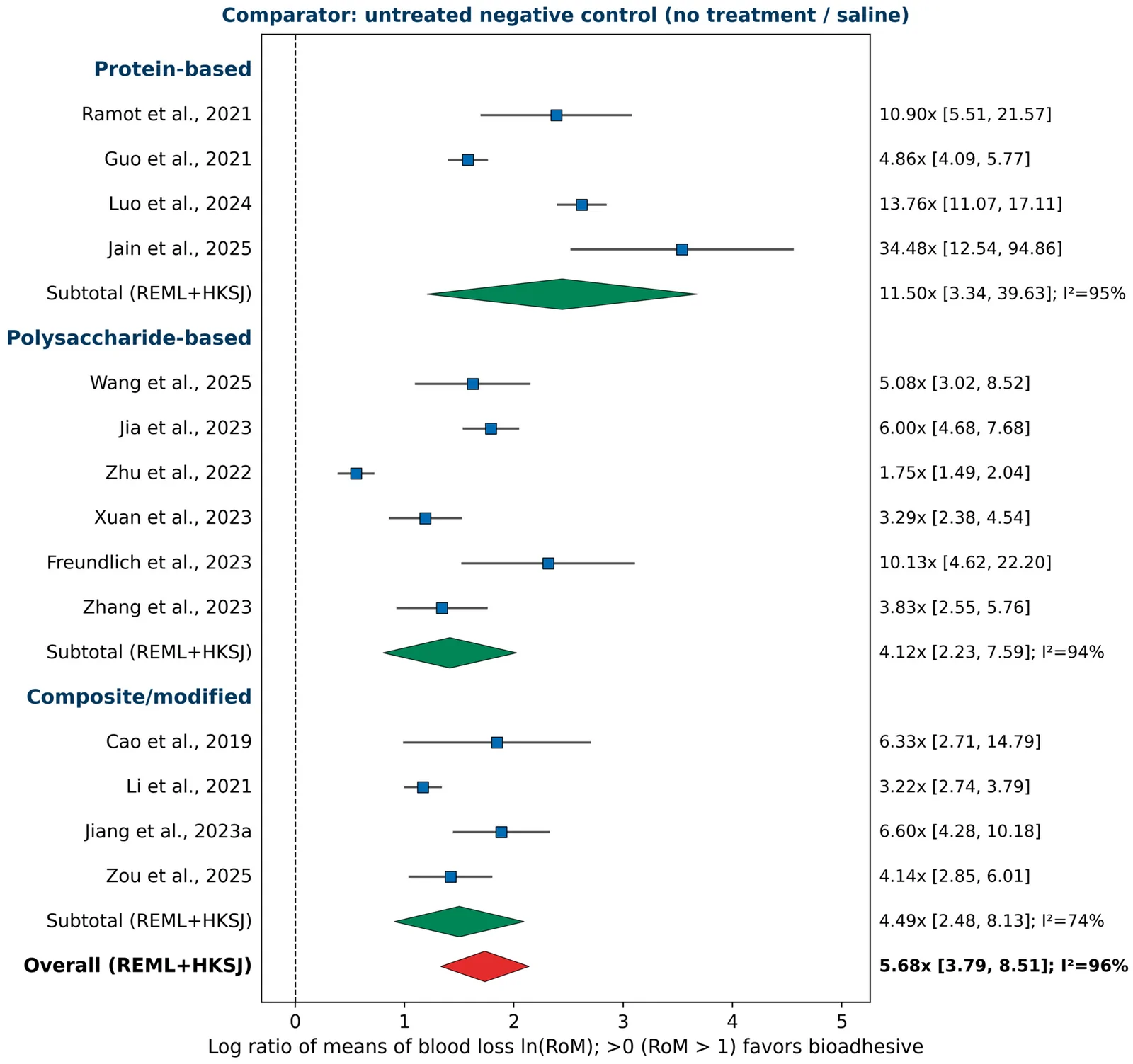
Forest plot of intraoperative blood loss (log ratio of means, ln[RoM]) stratified by material class: bioadhesive versus untreated negative control. Random-effects meta-analysis (REML + HKSJ): overall RoM = 5.68 (95% CI: 3.79 to 8.51), corresponding to a 5.68-fold reduction; *I*^2^ = 96%, *τ*^2^ = 0.358; subgroup meta-regression *p* = 0.064. Green diamonds represent subgroup pooled estimates; the red diamond represents the overall pooled estimate. Abbreviations: CI, confidence interval; HKSJ, Hartung–Knapp–Sidik–Jonkman; REML, restricted maximum likelihood; RoM, ratio of means [6,10,11,12,18,21,22,24,28,31,38,43,45,48].

**Figure 9:**
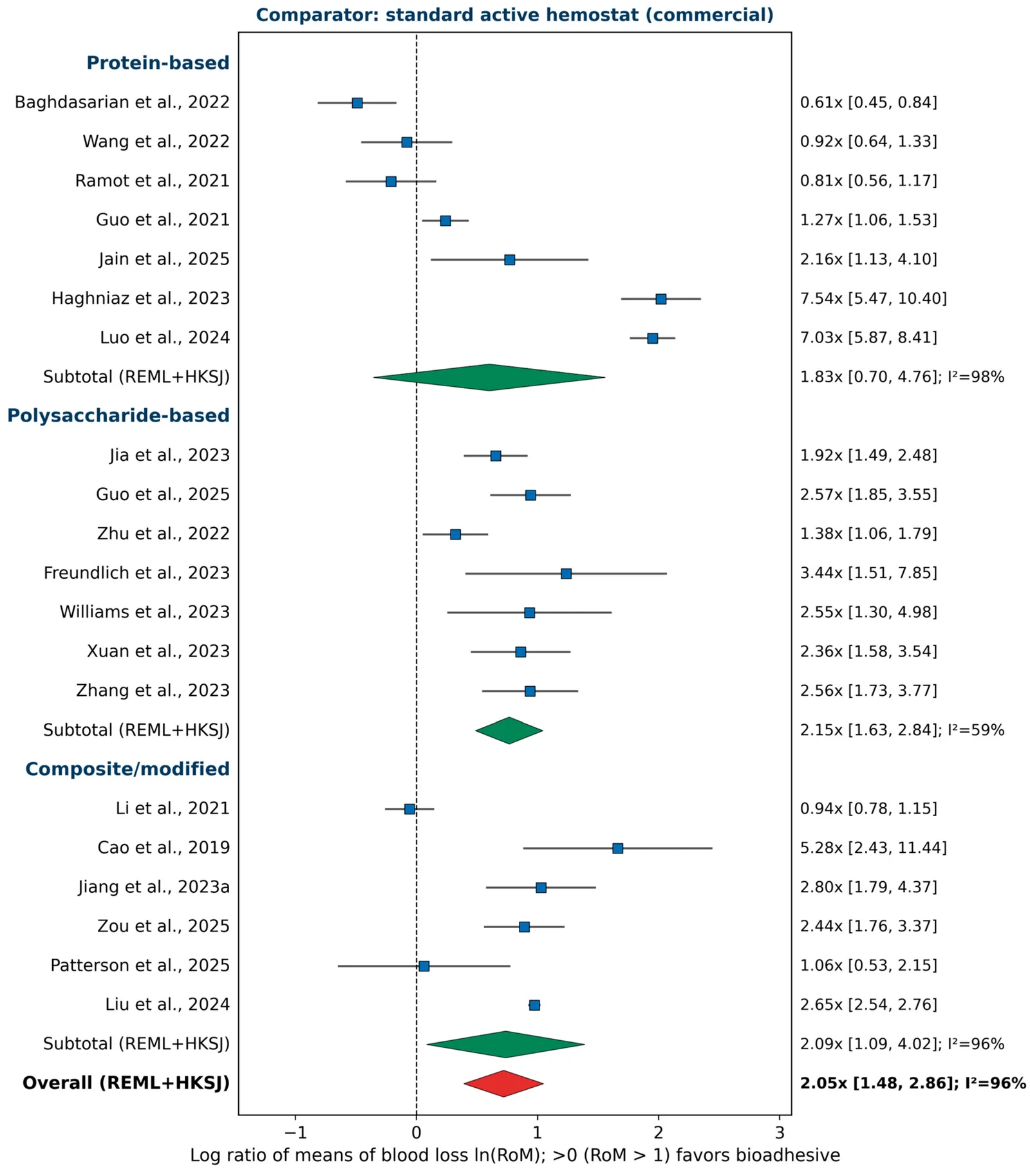
Forest plot of intraoperative blood loss (log ratio of means, ln[RoM]) stratified by material class: bioadhesive versus standard active hemostat. Random-effects meta-analysis (REML + HKSJ): overall RoM = 2.05 (95% CI: 1.48 to 2.86), corresponding to a 2.05-fold reduction; *I*^2^ = 96%, *τ*^2^ = 0.350; subgroup meta-regression *p* = 0.852. Green diamonds represent subgroup pooled estimates; the red diamond represents the overall pooled estimate. Abbreviations: CI, confidence interval; HKSJ, Hartung–Knapp–Sidik–Jonkman; REML, restricted maximum likelihood; RoM, ratio of means [5,6,9,10,11,12,18,21,22,24,28,29,30,31,38,42,43,44,46,48].

**Figure 10:**
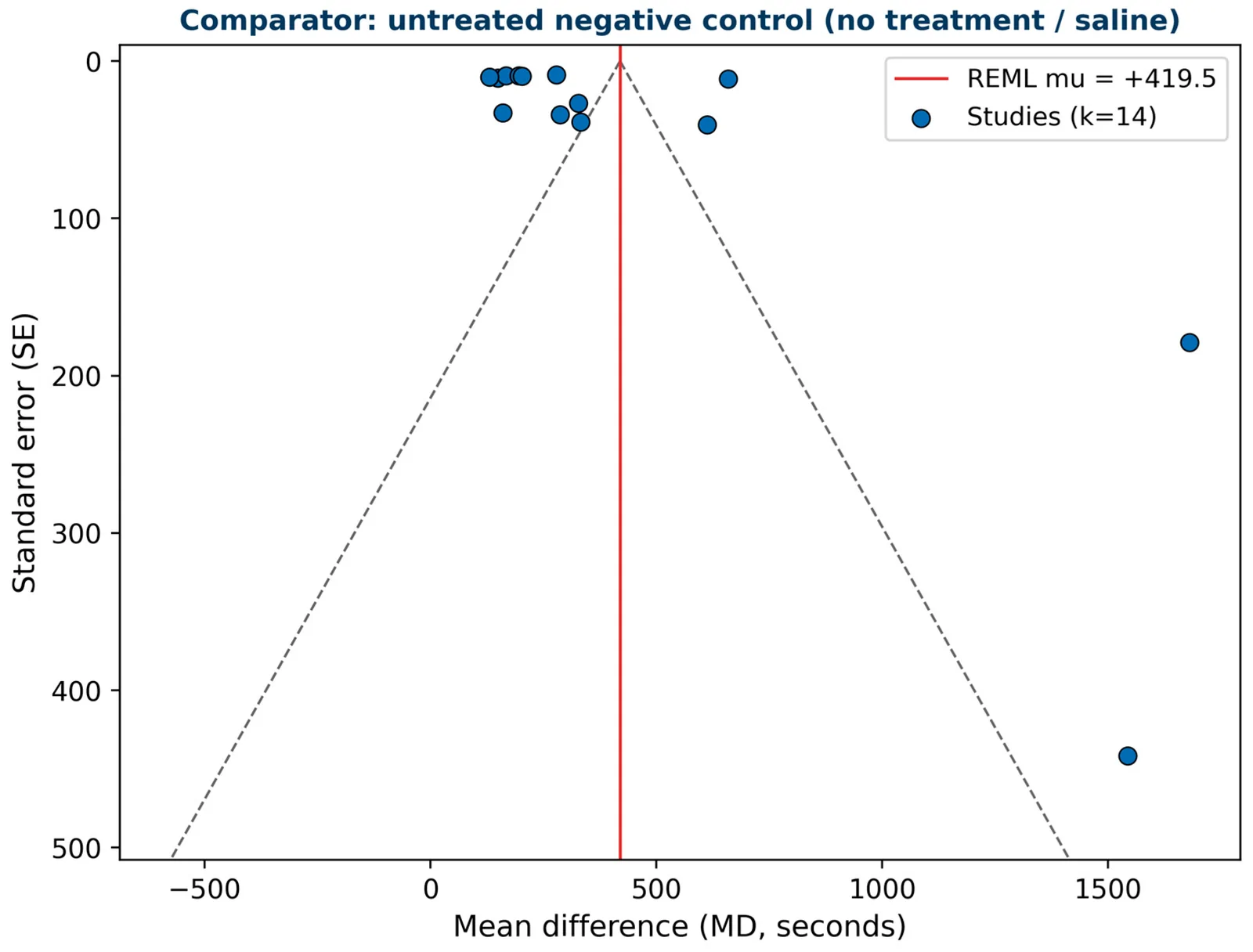
Funnel plot of effect size versus standard error for time to hemostasis: bioadhesive versus untreated negative control. The x-axis shows the raw mean difference (MD, seconds), and the y-axis shows the standard error (SE; inverted axis). The red vertical line marks the REML summary estimate (+419.5 s); dashed lines indicate the pseudo-95% confidence limits. Modified Egger test (1/√*N_total_*): *p* = 0.105. Abbreviations: MD, mean difference; REML, restricted maximum likelihood; SE, standard error.

**Figure 11:**
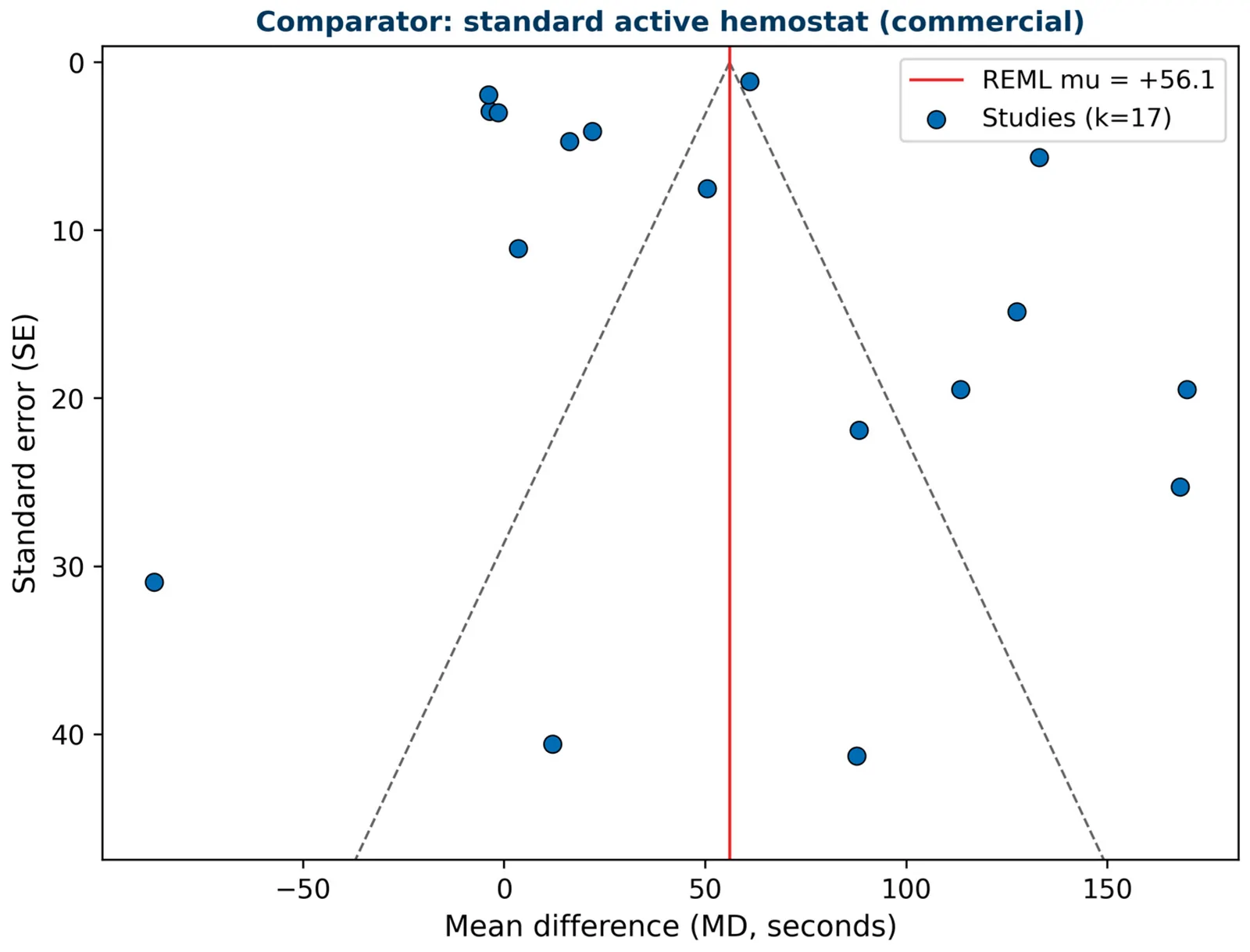
Funnel plot of effect size versus standard error for time to hemostasis: bioadhesive versus standard active hemostat. The x-axis shows the raw mean difference (MD, seconds), and the y-axis shows the standard error (SE; inverted axis). The red vertical line marks the REML summary estimate (+56.1 s); dashed lines indicate the pseudo-95% confidence limits. Modified Egger test (1/√*N_total_*): *p* = 0.240. Abbreviations: MD, mean difference; REML, restricted maximum likelihood; SE, standard error.

**Figure 12:**
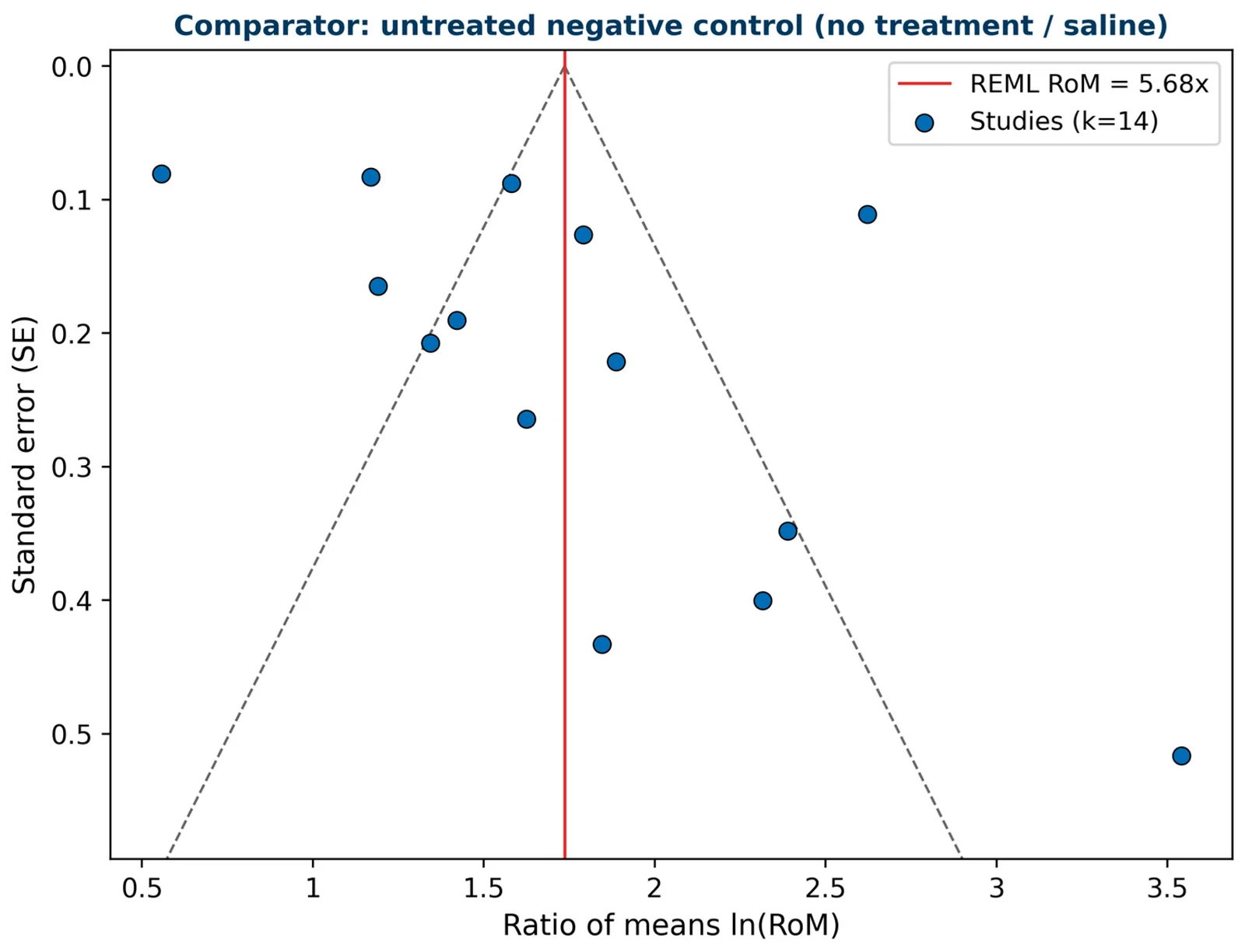
Funnel plot of effect size versus standard error for blood loss: bioadhesive versus untreated negative control. The x-axis shows the log ratio of means (ln[RoM]), and the y-axis shows the standard error (SE; inverted axis). The red vertical line marks the REML summary estimate (RoM = 5.68); dashed lines indicate the pseudo-95% confidence limits. Modified Egger test (1/√*N_total_*): *p* = 0.071. Abbreviations: REML, restricted maximum likelihood; RoM, ratio of means; SE, standard error.

**Figure 13:**
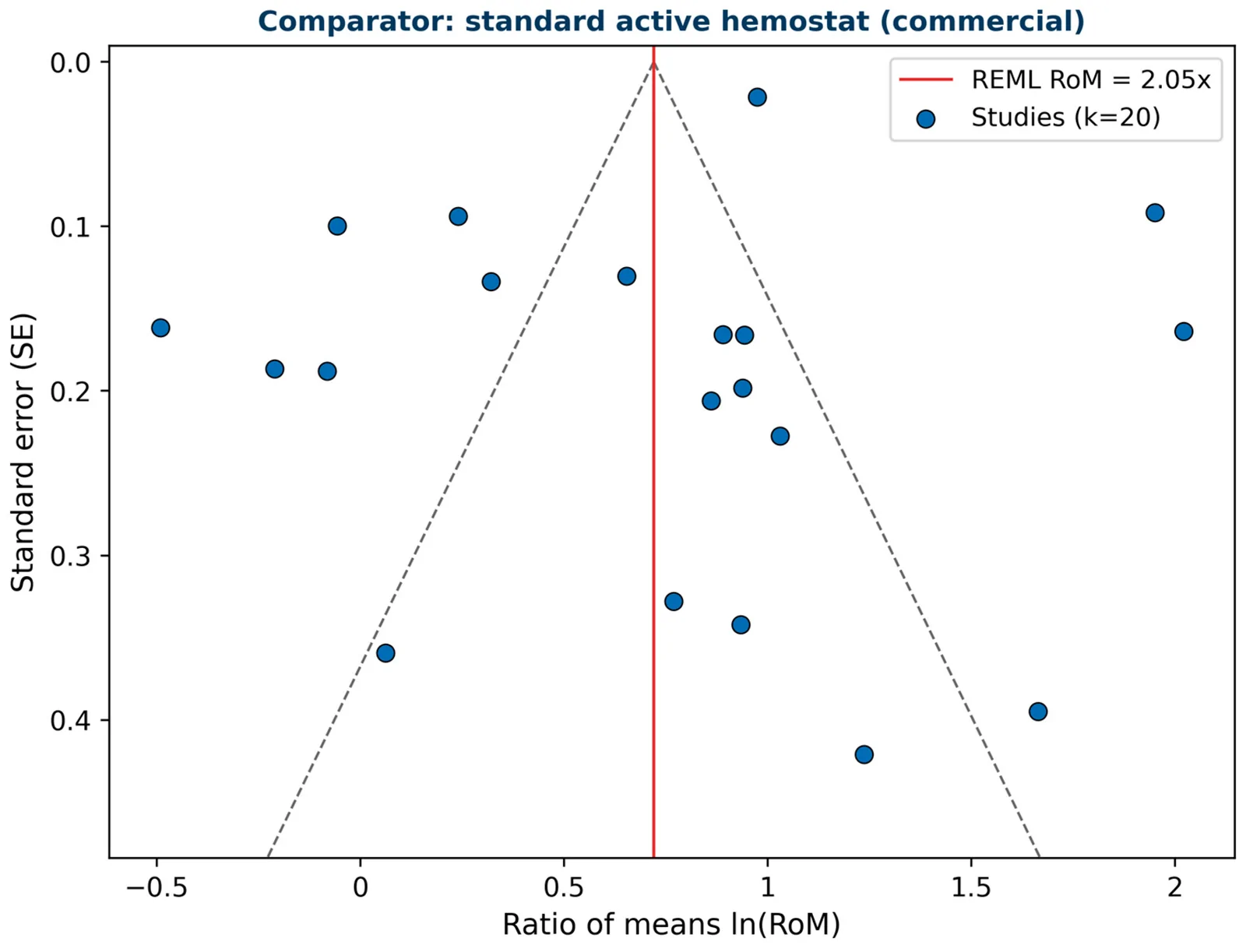
Funnel plot of effect size versus standard error for blood loss: bioadhesive versus standard active hemostat. The x-axis shows the log ratio of means (ln[RoM]), and the y-axis shows the standard error (SE; inverted axis). The red vertical line marks the REML summary estimate (RoM = 2.05); dashed lines indicate the pseudo-95% confidence limits. Modified Egger test (1/√*N_total_*): *p* = 0.989. Abbreviations: REML, restricted maximum likelihood; RoM, ratio of means; SE, standard error.

**Table 1:** Study eligibility criteria formulated according to the PICO framework.

PICO Component	Inclusion Criteria	Exclusion Criteria
Population (P)	Liver tissue: in vitro parenchymal models, preclinical in vivo animal models, and human patients undergoing hepatic surgery.	Non-hepatic tissue models or multi-organ investigations without segregated hepatic data.
Intervention (I)	Novel investigational bio-based formulations (tissue adhesives, sealants, or topical hemostatic matrices) containing predominantly biologically derived polymers (fibrin, collagen, gelatin, albumin, chitosan, alginate, cellulose, hyaluronic acid, pectin, etc.), evaluated as the experimental platform.	Purely synthetic surgical glues (e.g., unmodified cyanoacrylates, polyurethanes, acrylates) devoid of biological polymeric constituents.
Comparator (C)	Established commercial standard-of-care products (e.g., licensed commercial hemostats including oxidized regenerated/non-regenerated cellulose, collagen sponges, commercial fibrin sealants such as TachoSil), conventional surgical techniques (suture ligation, electrocautery, gauze packing), or untreated/saline negative controls, regardless of polymeric origin.	None.
Outcomes (O)	At least one quantifiable or qualifiable surgical outcome: hemostatic efficacy (time to hemostasis, blood loss), mechanical bonding/burst pressure, in vitro/in vivo biocompatibility, or clinical/postoperative outcomes.	Absence of quantitative or structured qualitative reporting on target surgical outcomes.
Study Design	Original primary research studies (in vitro mechanical/cytocompatibility assays, preclinical in vivo animal trials, and prospective or retrospective clinical investigations).	Narrative reviews, systematic reviews without primary data, conference abstracts, editorials, letters, or duplicate cohorts.
Language/Date	No language, geographical, or publication date restrictions (literature searched up to February 2026).	None.

Abbreviations: PICO, Population, Intervention, Comparator, and Outcomes.

**Table 2:** Baseline characteristics and methodological parameters of all included studies (*n* = 42).

No.	Study	Year	Study Design	Species/Model	Bioadhesive Formulation and Polymer Source
1	Anderson et al. [36]	2025	Veterinary RCT	Canine (client-owned dogs)	Adhesive gelatin-based sponge (mammalian gelatin)
2	Baghdasarian et al. [9]	2022	In vitro/in vivo	Rat (in vivo) and swine (ex vivo)	Dopamine-modified methacrylated gelatin hydrogel with Fe^3+^ coordination (GelMAC; porcine)
3	Barsky et al. [37]	2022	In vivo	Swine (penetrating trauma)	Ultra-low-viscosity sodium alginate injectable solution (algal origin)
4	Bochicchio et al. [15]	2025	Clinical RCT (Phase 1/2)	Human (partial hepatectomy)	Biodegradable fibrin sealant patch (sFilm-FS; human plasma-derived fibrinogen/thrombin)
5	Campos-Cuerva et al. [19]	2019	In vivo	Rat (liver biopsy)	Nanostructured human fibrin-agarose hybrid hydrogel (human plasma and marine agarose)
6	Cao et al. [38]	2019	In vitro/in vivo	Rat (liver transection)	Injectable carboxymethyl chitosan/gelatin/oxidized sodium alginate hydrogel (CMC-GEL/OSA)
7	Casado et al. [39]	2024	In vitro/in vivo	Rat (in vivo) & human (in vitro)	Cryopreserved nanostructured human fibrin-agarose hydrogel (human/algal origin)
8	Deineka et al. [40]	2021	In vitro/in vivo	Rat (partial resection)	Electrospun chitosan nanofibrous membrane (crustacean chitin derivative)
9	Du et al. [26]	2024	In vitro/in vivo	Rat (coagulopathic model)	Self-elastic phospholipid-modified chitosan sponge incorporating thrombin (crustacean/bovine)
10	Eskildsen et al. [16]	2023	In vitro/in vivo	Swine (punch biopsy)	Autologous whole blood-derived patch enriched with fibrin, platelets, and leukocytes
11	Fontes et al. [41]	2018	In vivo	Rat (parenchymal puncture)	Absorbable gelatin sponge (porcine) vs. equine collagen sponge
12	Freundlich et al. [28]	2023	In vitro/in vivo	Rat (liver laceration)	Bioadhesive microneedle patch array of Carbopol and chitosan with pullulan backing layer
13	Fujikawa et al. [17]	2025	Clinical Cohort	Human (robotic resection)	Microporous polysaccharide hemospheres (purified plant starch)
14	Gao et al. [3]	2023	In vivo	Rat (liver bleeding)	Hyaluronic acid functionalized with synthetic collagen- and vWF-binding peptides
15	Guo et al. [10]	2021	In vitro/in vivo	Rat (liver resection)	Visible-light crosslinked methacrylated gelatin hydrogel loaded with Bothrops atrox batroxobin (HAD)
16	Guo et al. [42]	2025	In vitro/in vivo	Rat, rabbit, swine	Injectable porous foam hydrogel based on sodium alginate and carboxymethyl chitosan (IHF)
17	Haghniaz et al. [29]	2023	In vitro/in vivo	Rat (liver laceration)	Methacrylated gelatin microneedle array reinforced with silicate nanosheets (GelMA-SN)
18	Jain et al. [21]	2025	In vitro/in vivo	Rat, swine	Naturally derived antimicrobial sealant: GelMA, dopamine methacrylamide, and pDDA (GelMAG)
19	Jia et al. [24]	2023	In vitro/in vivo	Rat, swine	Janus powder hemostat: phenylboronic acid/dopamine-modified alginate and hyaluronic acid (FJG)
20	Jiang et al. [27]	2023	In vitro/in vivo	Rat, rabbit	Adhesive hydrogel sponge with human umbilical cord MSC apoptotic extracellular vesicles (apoV-HS)
21	Jiang et al. [43]	2023	In vitro/in vivo	Rat, rabbit, canine	Chitosan lactate microparticles for non-compression hemostasis (Alaskan snow crab chitin)
22	Ko et al. [25]	2025	In vitro/in vivo	Rat (in vivo), canine (in vitro)	Adhesive flake particulates of calcium alginate, starch, and polyacrylamide ionic networks
23	Lake et al. [2]	2023	In vivo	Swine (liver biopsy punch)	Flowable microfibrillar bovine dermal collagen hemostat (Avitene paste)
24	Li et al. [11]	2021	In vitro/in vivo	Rat (partial hepatectomy)	Biomimetic hybrid hydrogel of oxidized hyaluronic acid, glycol chitosan, and stem cell secretome
25	Liu et al. [30]	2024	In vitro/in vivo	Rat (liver injury)	Hemostatic aerogel composed of TEMPO-oxidized cellulose nanofibers, fish collagen, and chitosan
26	Luo et al. [22]	2024	In vitro/in vivo	Rat (visceral bleeding)	Blue-laser crosslinked hemostatic peptide hydrogel based on GelMA and self-assembling peptides
27	MacDonald et al. [1]	2017	In vivo	Swine (liver abrasion/cut)	Oxidized regenerated cellulose hemostatic powder (plant-derived rayon cellulose)
28	Patterson et al. [5]	2025	In vivo	Swine (parenchymal cut)	Commercial chitosan dressings (ChitoSAM 100, Celox Rapid) and human fibrin patch
29	Ramot et al. [18]	2021	In vivo	Göttingen minipig	Biodegradable fibrin sealant patch on synthetic PEG/PCL/PLA polymer backing (sFilm-FS)
30	Slezak et al. [20]	2018	In vitro/in vivo	Rabbit (punch biopsy)	Bovine collagen patch coated with synthetic PEG NHS-ester crosslinker (Hemopatch)
31	Valle et al. [4]	2025	In vivo	Swine (stab trauma AAST V)	PEG-coated collagen patch (Hemopatch) vs. polysaccharide microspheres (PerClot)
32	Wang et al. [44]	2022	In vitro/in vivo	Rat, rabbit	Superabsorbent fish collagen sponge extracted from tilapia skin (Oreochromis niloticus)
33	Wang et al. [45]	2025	In vitro/in vivo	Rat (in vivo), swine (ex vivo)	Immunomodulatory hydrogel: oxidized dextran and ibuprofen-conjugated chitosan (ODA/CS-IBU)
34	Williams et al. [46]	2023	In vivo	Swine (solid organ trauma)	Biologic ‘Velcro’ tissue patch based on high-methoxyl citrus pectin (plant origin)
35	Xuan et al. [31]	2023	In vitro/in vivo	Mouse (liver incision)	Shape-memory cryogel based on chitosan and reduced graphene oxide (CTS cryogel)
36	Xue et al. [32]	2023	In vitro/in vivo	Rat (resection/defect)	Adhesive cryogel microparticles of poly(acrylic acid) crosslinked with chitosan (ACPs)
37	Zhang et al. [47]	2020	In vitro/in vivo	Mouse, rabbit	Thermosensitive hydrogel: catechol-modified chitosan, beta-glycerophosphate, and oyster peptides
38	Zhang et al. [35]	2021	Clinical RCT	Human (liver resection)	Oxidized non-regenerated cellulose gauze vs. oxidized regenerated cellulose gauze
39	Zhang et al. [12]	2023	In vitro/in vivo	Rat (liver laceration)	Injectable self-healing hydrogel of carboxymethyl chitosan and polyaldehyde dextran (CP100)
40	Zhao et al. [23]	2024	In vitro/in vivo	Rat (wedge resection)	Non-covalent crosslinked hydrogel based on chicken type II collagen and tannic acid (CT–5)
41	Zhu et al. [6]	2022	In vivo	Rat (liver puncture)	Biodegradable cellulose composite: sodium carboxymethyl cellulose and hydroxyethyl cellulose
42	Zou et al. [48]	2025	In vitro/in vivo	Rabbit (wedge resection)	Self-assembling powder of sodium alginate and porcine small intestinal submucosa ECM (APSO)

Abbreviations: ECM, extracellular matrix; PCL, polycaprolactone; PEG, polyethylene glycol; PLA, polylactic acid; RCT, randomized controlled trial; TEMPO, 2,2,6,6-tetramethylpiperidine–1-oxyl; vWF, von Willebrand factor.

**Table 3:** Methodological quality and risk-of-bias assessment across SYRCLE domains (preclinical animal studies, *n* = 38).

SYRCLE Risk-of-Bias Domain (*n* = 38 Animal Studies)	Low Risk	Unclear Risk	High Risk
Sequence generation (selection bias)	14 (36.8%)	24 (63.2%)	0 (0.0%)
Baseline characteristics (selection bias)	38 (100.0%)	0 (0.0%)	0 (0.0%)
Allocation concealment (selection bias)	0 (0.0%)	38 (100.0%)	0 (0.0%)
Random housing (performance bias)	0 (0.0%)	38 (100.0%)	0 (0.0%)
Blinding of caregivers/investigators (performance bias)	0 (0.0%)	5 (13.2%)	33 (86.8%)
Random outcome assessment (detection bias)	0 (0.0%)	38 (100.0%)	0 (0.0%)
Blinding of outcome assessors (detection bias)	8 (21.1%)	30 (78.9%)	0 (0.0%)
Incomplete outcome data (attrition bias)	38 (100.0%)	0 (0.0%)	0 (0.0%)
Selective outcome reporting (reporting bias)	38 (100.0%)	0 (0.0%)	0 (0.0%)
Other sources of bias	38 (100.0%)	0 (0.0%)	0 (0.0%)

Abbreviations: SYRCLE, Systematic Review Centre for Laboratory Animal Experimentation.

**Table 4:** Complete risk-of-bias assessment for human clinical and veterinary studies (RoB 2/ROBINS-I/adapted RoB 2).

Study	Design/Tool	Key Domain Concerns	Overall Judgment
Bochicchio et al., 2025 [15]	Human RCT/RoB 2	Open-label surgical outcome measurement (some concerns); other domains low	Some concerns
Zhang et al., 2021 [35]	Human RCT/RoB 2	Open-label surgical outcome measurement (some concerns); other domains low	Some concerns
Fujikawa et al., 2025 [17]	Retrospective cohort/ROBINS-I	Moderate confounding and non-blinded outcome measurement	Moderate risk of bias
Anderson et al., 2025 [36]	Veterinary RCT/RoB 2 (adapted)	High risk for outcome measurement (unblinded subjective bleeding scores); randomization low risk	High risk of bias (outcome measurement)

Abbreviations: RCT, randomized controlled trial; RoB 2, Cochrane Risk of Bias 2; ROBINS-I, Risk Of Bias In Non-randomized Studies of Interventions. Full domain-level ratings for all 38 preclinical animal studies (SYRCLE) are provided in Supplementary Table S1.

**Table 5:** Observed effect size ranges across material classes for primary outcomes (preclinical comparisons).

Outcome	Material Class	vs. Negative Control (*k*)	MD (s)/RoM (Fold Reduction)	vs. Active Comparator (*k*)	MD (s)/RoM (Fold Reduction)
Time to hemostasis	Protein-based	7	MD: +688.3 s [95% CI: 144.4 to 1232.3 s]	6	MD: +15.3 s [95% CI: −59.2 to +89.9 s]
Time to hemostasis	Polysaccharide-based	3	MD: +178.7 s [95% CI: 126.9 to 230.4 s]	6	MD: +71.0 s [95% CI: 4.2 to 137.8 s]
Time to hemostasis	Composite/modified	4	MD: +232.5 s [95% CI: 90.1 to 375.0 s]	5	MD: +85.8 s [95% CI: 2.0 to 169.6 s]
Intraoperative blood loss	Protein-based	4	RoM: 11.50× [95% CI: 3.34 to 39.63×]	7	RoM: 1.83× [95% CI: 0.70 to 4.76×]
Intraoperative blood loss	Polysaccharide-based	6	RoM: 4.12× [95% CI: 2.23 to 7.59×]	7	RoM: 2.15× [95% CI: 1.63 to 2.84×]
Intraoperative blood loss	Composite/modified	4	RoM: 4.49× [95% CI: 2.48 to 8.13×]	6	RoM: 2.09× [95% CI: 1.09 to 4.02×]

Negative control: untreated injury, blank, or saline/PBS, with no hemostatic agent applied. Active comparator: commercial topical hemostat or conventional surgical control (gauze, packing, or suture), as reported in the primary study. Abbreviations: CI, confidence interval; *k*, number of pairwise comparisons; MD, raw mean difference (seconds; MD > 0 favors the bioadhesive); RoM, ratio of means (RoM > 1 indicates a reduction favoring the bioadhesive).

**Table 6:** In vitro adhesive/shear strength (kPa) across material classes (intervention values; median (range)).

Material Class	Number of Studies	Median Shear Strength (kPa)	Range (kPa)
Protein-based (fibrin, collagen, gelatin)	5	42.0	33.7 to 104.9
Polysaccharide-based (chitosan, alginate, cellulose)	3	10.6	0.66 to 47.6
Composite/chemically modified	3	17.2	7.20 to 90.0

Data were compiled from 18 quantitative shear-strength measurements across 11 preclinical studies.

**Table 7:** Summary of primary clinical outcomes from included human and veterinary trials.

Study	Clinical Outcome	Treatment Group (Role)	*n*	Reported Value	Unit
Anderson et al., 2025 [36]	Time to hemostasis (T0)	Adhesive gelatin sponge (Intervention)	22	1.00	Bleeding score (0–5)
		Plain collagen sponge (Comparator)	23	1.90	
	Time to hemostasis (T2)	Adhesive gelatin sponge (Intervention)	22	0.50	
		Plain collagen sponge (Comparator)	23	1.40	
	Time to hemostasis (T5)	Adhesive gelatin sponge (Intervention)	22	0.10	
		Plain collagen sponge (Comparator)	23	1.00	
	Time to hemostasis (T10)	Adhesive gelatin sponge (Intervention)	22	0.10	
		Plain collagen sponge (Comparator)	23	0.70	
Bochicchio et al., 2025 [15]	Time to hemostasis	sFilm-FS fibrin patch (Intervention)	17	3.20 ± 2.67	min
		TachoSil collagen-fibrin (Comparator)	16	3.00 ± 0.00	
	Intraoperative blood loss	sFilm-FS fibrin patch (Intervention)	17	250.0 ± 327.6	mL
		TachoSil collagen-fibrin (Comparator)	16	139.4 ± 163.3	
	Postoperative drain volume	sFilm-FS fibrin patch (Intervention)	15	522.7 ± 532.5	mL
		TachoSil collagen-fibrin (Comparator)	14	97.0 ± 61.1	
Fujikawa et al., 2025 [17]	Intraoperative blood loss	No hemostat (Control)	47	Me: 0.0	mL
		Fibrin sealant patch (Comparator)	23	Me: 34.0	
		Microporous hemospheres (Intervention)	71	Me: 11.0	
	Postoperative complications	No hemostat (Control)	47	2.1%	incidence rate
		Fibrin sealant patch (Comparator)	23	8.7%	
		Microporous hemospheres (Intervention)	71	0.0%	
	Hospital length of stay	No hemostat (Control)	47	Me: 8.0	days
		Fibrin sealant patch (Comparator)	23	Me: 9.0	
		Microporous hemospheres (Intervention)	71	Me: 8.0	
	Hemostat application time	Fibrin sealant patch (Comparator)	23	Me: 382.0	s
		Microporous hemospheres (Intervention)	25	Me: 36.0	
Zhang et al., 2021 [35]	Time to hemostasis	Oxidized regenerated cellulose (Comparator)	38	238.8 ± 121.6	s
		Oxidized non-regenerated cellulose (Intervention)	37	193.7 ± 85.3	
	Drain volume (Postop Day 1)	Oxidized regenerated cellulose (Comparator)	38	Me: 68.0	mL
		Oxidized non-regenerated cellulose (Intervention)	37	Me: 75.0	
	Drain volume (Postop Day 2)	Oxidized regenerated cellulose (Comparator)	38	Me: 20.0	mL
		Oxidized non-regenerated cellulose (Intervention)	37	Me: 40.0	
	Hospital length of stay	Oxidized regenerated cellulose (Comparator)	38	Me: 17.0	days
		Oxidized non-regenerated cellulose (Intervention)	37	Me: 18.0	
	Postoperative recovery stay	Oxidized regenerated cellulose (Comparator)	38	Me: 9.0	days
		Oxidized non-regenerated cellulose (Intervention)	37	Me: 9.0	

Abbreviations: Me, median; min, minutes; mL, milliliters; *n*, sample size; s, seconds; SD, standard deviation.

**Table 8:** Summary meta-analysis results for primary outcomes (random-effects model, REML + HKSJ; preclinical studies only).

Outcome/Comparison	*k*	Raw Mean Difference MD (s) [95% CI]	Ratio of Means RoM [95% CI]	Hedges’ *g* [95% CI] (Sensitivity)	*p*-Value	*I*^2^ (%)
Time to hemostasis: bioadhesive vs. negative control	14	+419.53 [164.44; 674.62]	5.07× [3.10; 8.29]	+9.72 [5.90; 13.54]	*p* = 0.0035	99%
Time to hemostasis: bioadhesive vs. active comparator	17	+56.05 [20.14; 91.96]	1.91× [1.31; 2.79]	+3.30 [0.45; 6.14]	*p* = 0.0044	99%
Blood loss: bioadhesive vs. negative control	14	–	5.68× [3.79; 8.51]	+5.37 [3.65; 7.10]	*p* < 0.0001	96%
Blood loss: bioadhesive vs. active comparator	20	–	2.05× [1.48; 2.86]	+2.75 [0.74; 4.76]	*p* = 0.0002	96%

Negative control: untreated injury, blank, or saline/PBS, with no hemostatic agent applied. Active comparator: commercial topical hemostat or conventional surgical control (gauze, packing, or suture), as reported in the primary study. Abbreviations: CI, confidence interval; *k*, number of pairwise comparisons; MD, raw mean difference (seconds); RoM, ratio of means.

**Table 9:** Subgroup meta-analysis by biopolymer material class (REML + HKSJ).

Analysis/Subgroup	*k*	MD (s) [95% CI]	RoM [95% CI]	*I*^2^ (%)	Meta-Regression *p*-Value
Time to hemostasis vs. negative control:	14	+419.53 [164.44; 674.62]	5.07× [3.10; 8.29]	99%	*p* = 0.178
Protein-based	7	+688.31 [144.36; 1232.26]	7.18× [2.44; 21.14]	100%	—
Polysaccharide-based	3	+178.66 [126.89; 230.42]	3.72× [1.90; 7.28]	61%	—
Composite/modified	4	+232.54 [90.06; 375.02]	4.55× [1.76; 11.75]	95%	—
Time to hemostasis vs. active comparator:	17	+56.05 [20.14; 91.96]	1.91× [1.31; 2.79]	99%	*p* = 0.213
Protein-based	6	+15.34 [−59.17; 89.86]	1.46× [0.82; 2.61]	99%	—
Polysaccharide-based	6	+71.00 [4.19; 137.81]	2.18× [1.14; 4.19]	97%	—
Composite/modified	5	+85.84 [2.03; 169.65]	2.41× [1.18; 4.90]	99%	—
Blood loss vs. negative control:	14	—	5.68× [3.79; 8.51]	96%	*p* = 0.064
Protein-based	4	—	11.50× [3.34; 39.63]	95%	—
Polysaccharide-based	6	—	4.12× [2.23; 7.59]	94%	—
Composite/modified	4	—	4.49× [2.48; 8.13]	74%	—
Blood loss vs. active comparator:	20	—	2.05× [1.48; 2.86]	96%	*p* = 0.852
Protein-based	7	—	1.83× [0.70; 4.76]	98%	—
Polysaccharide-based	7	—	2.15× [1.63; 2.84]	59%	—
Composite/modified	6	—	2.09× [1.09; 4.02]	96%	—

Negative control: untreated injury, blank, or saline/PBS, with no hemostatic agent applied. Active comparator: commercial topical hemostat or conventional surgical control (gauze, packing, or suture), as reported in the primary study. Abbreviations: CI, confidence interval; *k*, number of pairwise comparisons; MD, raw mean difference (seconds); REML, restricted maximum likelihood; RoM, ratio of means.

**Table 10:** Classification of all quantitative meta-analysis interventions by primary hemostatic mechanism.

Primary Hemostatic Mechanism	Study (ID)	Intervention (Biomaterial Specification)
Physical absorption	Deineka et al., 2021 [40]	Electrospun chitosan nanofibrous membranes (ChEsM)
	Freundlich et al., 2023 [28]	Carbopol/chitosan microneedle patch array
	Liu et al., 2024 [30]	Porous TEMPO-oxidized nanocellulose/collagen/chitosan aerogel (TCNF/COL/CS)
	Wang et al., 2022 [44]	Superabsorbent fish collagen sponge
	Xuan et al., 2023 [31]	Porous shape-memory chitosan/reduced graphene oxide cryogel (CTS cryogel)
	Zhu et al., 2022 [6]	Composite porous carboxymethyl/hydroxyethyl cellulose sponge
Chemical adhesion	Baghdasarian et al., 2022 [9]	Catechol-modified methacrylated gelatin adhesive hydrogel (GelMAC)
	Jain et al., 2025 [21]	Photopolymerizable antimicrobial gelatin bioadhesive (GelMAG/DMA/pDDA)
	Jiang et al., 2023 [27]	Extracellular vesicle-functionalized hyaluronic acid sponge (apoV-HS)
	Li et al., 2021 [11]	Glycol chitosan/oxidized hyaluronic acid biomimetic hydrogel (HC Hydrogel)
	Wang et al., 2025 [45]	Oxidized dextran and ibuprofen-conjugated chitosan matrix (ODA/CS-IBU)
	Williams et al., 2023 [46]	Biologic ‘Velcro’ tissue patch based on high-methoxyl pectin
	Zhang et al., 2023 [12]	Carboxymethyl chitosan/polyaldehyde dextran hydrogel (CP100)
	Zou et al., 2025 [48]	Self-assembling alginate/small intestinal submucosa ECM powder (APSO)
Coagulation activation	Eskildsen et al., 2023 [16]	Autologous whole blood-derived fibrin/platelet patch
	Patterson et al., 2025 [5]	Hemostatic chitosan gauze (ChitoSAM 100)
	Ramot et al., 2021 [18]	Biodegradable fibrin sealant patch (sFilm-FS)
Combined mechanism	Cao et al., 2019 [38]	Carboxymethyl cellulose/gelatin/oxidized sodium alginate hydrogel (CMC-GEL/OSA)
	Guo et al., 2021 [10]	Gelatin–batroxobin visible-light-crosslinked adhesive hydrogel (HAD)
	Guo et al., 2025 [42]	Injectable porous alginate/chitosan foam hydrogel (IHF)
	Haghniaz et al., 2023 [29]	GelMA microneedle array with silicate nanosheets (GelMA-SN)
	Jia et al., 2023 [24]	Janus powder hemostat: phenylboronic acid/alginate/hyaluronic acid (FJG)
	Ko et al., 2025 [25]	Adhesive calcium alginate/starch/polyacrylamide flake particulates (A5S5-PC)
	Luo et al., 2024 [22]	Blue-laser-crosslinked GelMA/peptide hydrogel (HPH)
	Zhang et al., 2020 [47]	Thermosensitive catechol-chitosan/beta-glycerophosphate hydrogel (CS-C/OP/β-GP)

Assignments were confirmed by independent review of formulation architecture and the stated mechanism of action.

**Table 11:** Subgroup meta-analysis by primary hemostatic mechanism (REML + HKSJ).

Hemostatic Mechanism/Analysis	*k*	Time to Hemostasis MD (s) [95% CI]	Blood Loss RoM [95% CI]	*I*^2^ (%)	Meta-Regression *p*-Value
Time to hemostasis vs. active comparator	17	+56.05 [20.14; 91.96]	1.91× [1.31; 2.79]	99%	*p* = 0.082
Coagulation activation	2	+4.0 [−131.9; 139.9]	1.06× [0.45; 2.50]	0%	–
Chemical adhesion	4	+24.8 [−116.1; 165.8]	1.41× [0.68; 2.92]	97%	–
Physical absorption	5	+29.8 [−18.9; 78.4]	1.45× [0.82; 2.56]	100%	–
Combined mechanism	6	+113.9 [49.2; 178.5]	2.74× [1.52; 4.94]	98%	–
Blood loss vs. active comparator	20	–	2.05× [1.48; 2.86]	96%	*p* = 0.090
Coagulation activation	2	–	0.86× [0.10; 7.03]	0%	–
Chemical adhesion	7	–	1.73× [0.97; 3.08]	92%	–
Physical absorption	5	–	1.87× [0.98; 3.54]	93%	–
Combined mechanism	6	–	3.42× [1.54; 7.57]	98%	–

Negative control: untreated injury, blank, or saline/PBS, with no hemostatic agent applied. Active comparator: commercial topical hemostat or conventional surgical control (gauze, packing, or suture), as reported in the primary study. Abbreviations: CI, confidence interval; *k*, number of comparisons; MD, raw mean difference (seconds); RoM, ratio of means.

**Table 12:** Mixed-effects meta-regression evaluating the contribution of study covariates to heterogeneity.

Analytical Pool	Covariate (Moderator)	Slope Coefficient (*β*)	Omnibus *Q_M_* (*p*-Value)	Explained Variance (*R*^2^)
Time to hemostasis vs. negative control	Species (rodents vs. large animals)	−2.84	0.560	0%
Time to hemostasis vs. negative control	Injury geometry (resection vs. local defect)	−4.97	0.186	3%
Time to hemostasis vs. active comparator	Species (rodents vs. large animals)	+2.75	0.449	0%
Time to hemostasis vs. active comparator	Injury geometry (resection vs. local defect)	−2.43	0.435	0%
Blood loss vs. negative control	Species (rodents vs. large animals)	+3.17	0.109	12%
Blood loss vs. negative control	Injury geometry (resection vs. local defect)	+0.50	0.763	0%
Blood loss vs. active comparator	Species (rodents vs. large animals)	+2.49	0.229	0%
Blood loss vs. active comparator	Injury geometry (resection vs. local defect)	−2.53	0.185	0%

Negative control: untreated injury, blank, or saline/PBS, with no hemostatic agent applied. Active comparator: commercial topical hemostat or conventional surgical control (gauze, packing, or suture), as reported in the primary study. Abbreviations: *Q_M_*, omnibus test of moderators; *R*^2^, percentage of between-study variance explained; REML, restricted maximum likelihood; *β*, regression slope coefficient.

**Table 13:** Three-part sensitivity analysis assessing methodological robustness, blinded outcome assessment, and iterative leave-one-out analysis.

Analytical Pool/Outcome	Baseline Pooled Effect (*k*)	High-RoB Excluded (*k*)	Blinded Outcome Assessment Only (*k*)	Leave-One-Out Range & Stability
Time to hemostasis vs. negative control	MD = +419.53 s (*g* = +8.63) [*k* = 14]	*g* = +9.24 [95% CI: 5.66 to 12.83] [*k* = 13]	Single study (*k* = 1, Deineka et al. [40]: *g* = +13.56); meta-analytic pooling precluded	MD = +305.63 s to +445.68 s (all *p* < 0.01; High stability)
Time to hemostasis vs. active comparator	MD = +56.05 s (*g* = +2.59) [*k* = 17]	*g* = +2.84 [95% CI: 0.10 to 5.59] [*k* = 16]	*g* = +1.29 [95% CI: −6.31 to +8.90], *p* = 0.541 [*k* = 3: Deineka et al. [40], Eskildsen et al. [16], Guo et al. [42]]	MD = +48.95 s to +63.61 s (all *p* < 0.01; High stability)
Blood loss vs. negative control	RoM = 5.68x (*g* = +4.60) [*k* = 14]	*g* = +4.99 [95% CI: 3.13 to 6.86] [*k* = 12]	Single study (*k* = 1, Deineka et al. [40]: *g* = +4.87); meta-analytic pooling precluded	RoM = 5.10× to 6.17× (all *p* < 0.001; High stability)
Blood loss vs. standard hemostat	RoM = 2.05x (*g* = +2.40) [*k* = 20]	*g* = +2.67 [95% CI: 0.60 to 4.74] [*k* = 17]	*g* = +4.43 [95% CI: −10.50 to +19.37], *p* = 0.283 [*k* = 2: Eskildsen et al. [16], Deineka et al. [40]]	RoM = 1.92× to 2.20× (all *p* < 0.001; High stability)

Negative control: untreated injury, blank, or saline/PBS, with no hemostatic agent applied. Active comparator: commercial topical hemostat or conventional surgical control (gauze, packing, or suture), as reported in the primary study. Abbreviations: *k*, number of pairwise comparisons; MD, raw mean difference; RoM, ratio of means.

**Table 14:** Statistical assessment of funnel plot asymmetry using the modified sample-size-based Egger regression test.

Analytical Pool/Outcome	*k*	Precision Predictor	Modified Egger Test *t* (*df*)	*p*-Value
Time to hemostasis vs. negative control	14	1/√*N_total_*	*t* = −1.75 (*df* = 12)	*p* = 0.1049
Time to hemostasis vs. active comparator	17	1/√*N_total_*	*t* = 1.22 (*df* = 15)	*p* = 0.2399
Blood loss vs. negative control	14	1/√*N_total_*	*t* = 1.99 (*df* = 12)	*p* = 0.0705
Blood loss vs. active comparator	20	1/√*N_total_*	*t* = −0.01 (*df* = 18)	*p* = 0.9893

Negative control: untreated injury, blank, or saline/PBS, with no hemostatic agent applied. Active comparator: commercial topical hemostat or conventional surgical control (gauze, packing, or suture), as reported in the primary study. Abbreviations: *df*, degrees of freedom; *k*, number of pairwise comparisons; *N_total_*, total sample size; *t*, Student’s t-statistic.

**Table 15:** Certainty-of-evidence profile for primary outcomes according to the adapted GRADE framework.

Outcome/Comparison	*k*	Pooled Effect Estimate (REML + HKSJ)	95% Prediction Interval (PI)	Domain-by-Domain Downgrading Rationales	GRADE Certainty
Time to hemostasis vs. active comparator	17	MD = +56.05 s [20.14; 91.96]/RoM = 1.91×	−90.37 to +202.47 s (crosses null)	Risk of bias (−1): lack of blinding in 79% and unclear allocation concealment in 100%; inconsistency (−1): extreme heterogeneity (*I*^2^ = 99%) and PI crosses null; indirectness (−1): young healthy animal models vs. comorbid human liver parenchyma	Very Low
Time to hemostasis vs. negative control	14	MD = +419.53 s [164.44; 674.62]/RoM = 5.07×	−617.91 to +1456.97 s (crosses null)	Risk of bias (−1): pervasive lack of allocation concealment and assessor blinding; inconsistency (−1): extreme heterogeneity (*I*^2^ = 99%) and PI crosses null; indirectness (−1): young healthy animal models vs. diseased human livers	Very Low
Blood loss vs. active comparator	20	RoM = 2.05× [1.48; 2.86] fold reduction	0.48× to 8.74× fold reduction (crosses null)	Risk of bias (−1): unclear allocation concealment and lack of blinding; inconsistency (−1): high heterogeneity (*I*^2^ = 96%) and PI crosses null; indirectness (−1): healthy animal models vs. human surgical setting	Very Low
Blood loss vs. negative control	14	RoM = 5.68× [3.79; 8.51] fold reduction	1.35× to 23.88× fold reduction	Risk of bias (−1): unclear allocation concealment and lack of blinding; inconsistency (−1): high heterogeneity (*I*^2^ = 96%); indirectness (−1): healthy animal models vs. human surgical setting	Low

Negative control: untreated injury, blank, or saline/PBS, with no hemostatic agent applied. Active comparator: commercial topical hemostat or conventional surgical control (gauze, packing, or suture), as reported in the primary study. Abbreviations: GRADE, Grading of Recommendations Assessment, Development, and Evaluation; HKSJ, Hartung–Knapp–Sidik–Jonkman; MD, raw mean difference; REML, restricted maximum likelihood; RoM, ratio of means.

## Data Availability

The data presented in this study are available within the article and its Supplementary Materials. The raw extraction tables and analytical code are available from the corresponding author upon reasonable request.

## Supplementary Materials

The following supporting information can be downloaded at: https://www.mdpi.com/article/10.3390/jfb17100511/s1, Document S1: Study protocol; Document S2: Search strategies and full queries for PubMed, Dimensions, Consensus, and SciSpace; Document S3: Master data extraction sheet of included studies and outcomes; Table S1: Study-by-domain SYRCLE risk-of-bias matrix for preclinical/in vivo animal assessments.

## Abbreviations

The following abbreviations are used in this manuscript:

AAST	American Association for the Surgery of Trauma
ACPs	Adhesive Cryogel Particles
AI	Artificial Intelligence
ALT	Alanine Aminotransferase
apoV-HS	Apoptotic Vesicle-Encapsulated Hydrogel Sponge
APSO	Alginate/Small Intestinal Submucosa ECM Powder
ARRIVE	Animal Research: Reporting of In Vivo Experiments
AST	Aspartate Aminotransferase
BL	Blood Loss
ChEsM	Chitosan Electrospun Membrane
CI	Confidence Interval
CMC	Carboxymethyl Cellulose
DAMPs	Damage-Associated Molecular Patterns
DL	DerSimonian–Laird
ECM	Extracellular Matrix
FJG	Janus Powder Hemostat (Phenylboronic acid/Alginate/Hyaluronic acid)
GelMA	Gelatin Methacryloyl
GelMAC	Catechol-Functionalized Gelatin Methacryloyl
GRADE	Grading of Recommendations Assessment, Development, and Evaluation
HAD	Gelatin–Batroxobin Adhesive Hydrogel
HKSJ	Hartung–Knapp–Sidik–Jonkman
HPH	Hemostatic Peptide Hydrogel
HUVEC	Human Umbilical Vein Endothelial Cell
IHF	Injectable Hemostatic Foam
IRI	Ischemia–Reperfusion Injury
LOO	Leave-One-Out
MD	Mean Difference
MSC	Mesenchymal Stem Cell
PCL	Polycaprolactone
PEG	Polyethylene Glycol
PICO	Population, Intervention, Comparator, and Outcomes
PI	Prediction Interval
PLA	Polylactic Acid
PRISMA	Preferred Reporting Items for Systematic Reviews and Meta-Analyses
RCT	Randomized Controlled Trial
REML	Restricted Maximum Likelihood
RoB 2	Cochrane Risk of Bias 2
ROBINS-I	Risk Of Bias In Non-randomized Studies—of Interventions
RoM	Ratio of Means
SD	Standard Deviation
SE	Standard Error
SPOT GRADE	Surface Bleeding Severity Scale
SYRCLE	Systematic Review Centre for Laboratory animal Experimentation
TEMPO	2,2,6,6-Tetramethylpiperidine-1-oxyl
TTH	Time to Hemostasis
VIBe	Validation of Intraoperative Bleeding
vWF	von Willebrand Factor
