# Supplementary material for: Bio-Based Hemostatic Adhesives, Sealants, and Local Hemostatic Agents for Liver Tissue: A Systematic Review with Meta-Analysis of Preclinical Data
Source: J Funct Biomater. 2026 Oct 11;17(10):511. doi: 10.3390/jfb17100511 (PMC13706002; doi:10.3390/jfb17100511)
Supplement: Supplementary file 1 [file jfb-17-00511-s001.zip › S1_Study_protocol.pdf]

SYSTEMATIC REVIEW PROTOCOL

1. TITLE

Bio-based hemostatic adhesives, sealants, and local hemostatic agents for liver tissue: a systematic review with meta-analysis of preclinical databio-based adhesives for liver sealing: a systematic review of preclinical and clinical studies.

2. ADMINISTRATIVE INFORMATION

- Authors: (List the full names and affiliations of all authors)
- Corresponding author: (Provide the email address of the corresponding author)
- Author contributions: (Briefly describe each author's contribution to the development of this protocol)
- Funding sources: (Indicate funding sources, if any)
- Conflicts of interest: (Declare the absence or presence of conflicts of interest)
- Protocol registration: This protocol will be registered Open Science Network.

3. INTRODUCTION

Rationale:
Surgical interventions on the liver (resections, trauma repair, transplantation) are associated with a high risk of bleeding and bile leakage. Hepatic parenchyma is friable and highly vascularized, which makes conventional hemostatic techniques (suturing, coagulation) insufficiently effective and potentially traumatic. Bio-based adhesives represent a promising alternative or adjunct to standard methods, with the potential to provide rapid hemostasis, seal bile ducts, and reduce the risk of postoperative complications.

Despite the existence of numerous bioadhesives (fibrin-, collagen-, gelatin-, albumin-, and polysaccharide-based, among others), evidence on their efficacy, optimal mechanical properties, and biocompatibility specifically for liver tissue requires systematization. This systematic review aims to synthesize and appraise available evidence from in vitro, preclinical (in vivo), and clinical studies in order to evaluate the current status and prospects of bioadhesives in liver surgery.

4. PRIMARY REVIEW QUESTION (PICO format)

- Population: Liver tissue (in vitro models, animal models, and humans).
- Intervention: Application of bio-based adhesives (synthesized or obtained by extraction) to liver tissue.
- Comparison: Comparison with other types of bioadhesives, standard surgical techniques (suturing, coagulation, hemostatic sponges), or no intervention / placebo.
- Outcomes:
    - Primary outcomes:
        1. Sealing/hemostatic efficacy: time to hemostasis; seal integrity (pressure resistance, including bile-duct sealing).
        2. Adhesive strength: tensile strength, shear strength, and other relevant mechanical parameters specific to the liver.
    - Secondary outcomes:
        1. Biocompatibility: degree of inflammatory response, cytotoxicity, biodegradation rate, fibrous capsule formation, and effects on liver regeneration.

2. Clinical outcomes (for human studies): incidence of postoperative complications (bleeding, bile leakage, biloma, abscess), operative time, intraoperative blood loss, and length of hospital stay.

5. METHODS

Inclusion and exclusion criteria:

Component	Inclusion criteria	Exclusion criteria
**Study types**	Any original studies (in vitro, in vivo animal, or human) evaluating the application of bioadhesives to the liver.	Review articles, letters to the editor, editorials, case reports, and dissertations (if the main results have been published as an article, the article will be included).
**Population**	Liver model	Studies on other organs unless separate liver-specific data are reported.
**Intervention**	Application of any adhesive whose primary component is of biological origin (e.g., fibrin, collagen, gelatin, albumin, chitosan, alginate, and others).	Use of fully synthetic adhesives (e.g., cyanoacrylates) unless used in combination with a biological component.
**Outcomes**	The study must report at least one outcome of interest: sealing efficacy, mechanical properties, or biocompatibility.	Studies that do not provide quantitative or qualitative data on the outcomes of interest.
**Language**	No language restrictions (with subsequent translation if necessary).	—
**Publication date**	No publication-date restrictions.	—

Information sources and search strategy:

A systematic search will be conducted in the following electronic databases/platforms:

- PubMed
- Google Scholar (first 200 results)
- Dimensions
- AI-based scholarly literature search services

In addition, a manual search of the reference lists of included studies and relevant review articles will be performed.

Search strategy for PubMed:

```
(
  liver[tiab] OR hepatic[tiab] OR hepatectom*[tiab]
)
```

AND

(

adhesiv*[tiab] OR glue*[tiab] OR "biological glue"[tiab] OR "bio glue"[tiab] OR bioadhesive*[tiab] OR bioglue[tiab] OR "surgical sealant"[tiab] OR "surgical sealants"[tiab] OR "tissue sealant"[tiab] OR "tissue sealants"[tiab] OR "hemostatic agent"[tiab] OR "hemostatic agents"[tiab] OR "haemostatic agent"[tiab] OR "haemostatic agents"[tiab] OR "fibrin glue"[tiab] OR "fibrin sealant"[tiab] OR "fibrin adhesive"[tiab] OR gelatin[tiab] OR albumin[tiab] OR chitosan[tiab] OR alginate[tiab] OR collagen[tiab] OR hydrogel*[tiab] OR "crosslinked gelatin"[tiab] OR "natural adhesive"[tiab] OR "natural adhesives"[tiab] OR "protein adhesive"[tiab] OR "polysaccharide adhesive"[tiab] OR "mussel inspired"[tiab] OR "marine derived"[tiab] OR "plant derived"[tiab]

)

AND

(

adhes*[tiab] OR bond*[tiab] OR seal*[tiab] OR hemosta*[tiab] OR haemosta*[tiab] OR strength*[tiab] OR tensil*[tiab] OR "shear strength"[tiab] OR "burst pressure"[tiab] OR "peel strength"[tiab] OR leak*[tiab] OR airtight*[tiab] OR watertight*[tiab] OR biocompatib*[tiab] OR cytotoxic*[tiab] OR degrad*[tiab] OR inflammat*[tiab] OR "tissue response"[tiab] OR "mechanical propert*"[tiab] OR "adhesive strength"[tiab] OR "bonding strength"[tiab] OR "time to hemostasis"[tiab] OR "bleeding time"[tiab] OR "blood loss"[tiab] OR "tissue healing"[tiab] OR "wound closure"[tiab] OR biocompatibility[tiab] OR biodegradation[tiab] OR "tissue regeneration"[tiab]

)

NOT

(

cyanoacrylate*[tiab] OR "synthetic adhesive"[tiab] OR "synthetic adhesives"[tiab] OR "synthetic polymer"[tiab] OR "synthetic polymers"[tiab] OR polyurethane[tiab] OR "acrylic adhesive"[tiab] OR "acrylic adhesives"[tiab]

)

NOT

(

kidney[tiab] OR renal[tiab] OR spleen[tiab] OR splenic[tiab] OR lung[tiab] OR pulmonary[tiab] OR pancreas[tiab] OR pancreatic[tiab] OR skin[tiab] OR cutaneous[tiab] OR dermal[tiab] OR dermis[tiab] OR epidermis[tiab] OR bone[tiab] OR cardiac[tiab] OR vascular[tiab] OR dental[tiab] OR orthodontic[tiab] OR endodontic[tiab] OR periodontal[tiab] OR tooth[tiab] OR teeth[tiab] OR "wound healing"[tiab] OR "wound repair"[tiab] OR scar[tiab] OR cicatrix[tiab] OR "burn wound"[tiab] OR "skin ulcer"[tiab] OR "pressure ulcer"[tiab] OR ulcer[tiab] OR "cutaneous wound"[tiab] OR keloid[tiab] OR "scar formation"[tiab] OR post-acne[tiab] OR "acne scars"[tiab]

)

Search strategy for Dimensions:

(

liver OR hepatic OR hepatectomy

```
)

AND

(

  adhesive OR glue OR biological glue OR bio glue OR bioadhesive OR bioglue OR "surgical sealant" OR "tissue sealant" OR "hemostatic agent" OR "haemostatic agent" OR "fibrin glue" OR "fibrin sealant" OR "fibrin adhesive" OR gelatin OR albumin OR chitosan OR alginate OR collagen OR hydrogel OR "crosslinked gelatin" OR "natural adhesive" OR "protein adhesive" OR "polysaccharide adhesive" OR "mussel inspired" OR "marine derived" OR "plant derived"

)

AND

(

  adhesi OR bond OR seal OR hemosta OR haemosta OR strength OR tensile OR "shear strength" OR "burst pressure" OR "peel strength" OR leak OR airtight OR watertight OR biocompatib OR cytotox OR degrad OR inflammat OR "tissue response" OR "mechanical propert" OR "adhesive strength" OR "bonding strength" OR "time to hemostasis" OR "bleeding time" OR "blood loss" OR "tissue healing" OR "wound closure" OR biocompatibility OR biodegradation OR "tissue regeneration"

)

NOT

(

  cyanoacrylate OR "synthetic adhesive" OR "synthetic polymer" OR polyurethane OR "acrylic adhesive"

)

NOT

(

  kidney OR renal OR spleen OR splenic OR lung OR pulmonary OR pancreas OR pancreatic OR skin OR cutaneous OR dermal OR dermis OR epidermis OR bone OR cardiac OR vascular OR dental OR orthodontic OR endodontic OR periodontal OR tooth OR teeth OR "wound healing" OR "wound repair" OR scar OR cicatrix OR "burn wound" OR "skin ulcer" OR "pressure ulcer" OR ulcer OR "cutaneous wound" OR keloid OR "scar formation" OR post-acne OR "acne scars"

)
```

Search strategy for Google Scholar:

```
liver hepatectomy biological adhesive sealant gelatin chitosan collagen alginate fibrin hemostasis mechanical properties biocompatibility -cyanoacrylate -synthetic
```

Query for Consensus:

```
What is the effectiveness and safety of biological adhesives for achieving hemostasis and sealing liver tissues compared to standard surgical methods or other bio adhesives, according to in vitro, preclinical and clinical studies?
```

Query for SciSpace:

```
Act as a methodological lead and do a systematic literature review on "biological adhesives and sealants for liver tissue: efficacy, mechanical properties, and biocompatibility in preclinical and clinical studies" (no date restrictions). Search PubMed, Web of Science, Scopus, and Google Scholar comprehensively. Inclusion criteria: original studies (in vitro, animal, human) evaluating biological adhesives/sealants (e.g., fibrin-, gelatin-, collagen-, chitosan-, alginate-based, natural hydrogels, mussel-inspired, or plant-derived materials) applied to liver tissue. Outcomes of interest include hemostasis time, sealing/leak pressure, adhesive/tensile/shear strength, biocompatibility (inflammatory response, cytotoxicity), biodegradation, and clinical outcomes (e.g., postoperative complications, bile leak, blood loss). Exclusion criteria: synthetic adhesives (cyanoacrylates, polyurethanes, acrylics); studies focused on other organs (kidney, spleen, lung, pancreas, skin, bone, cardiovascular, dental, or general wound healing); non-research publications (reviews, editorials, case reports without original data). Deliver: A PRISMA flow diagram of the study selection process. Risk-of-bias tables using: SYRCLE's Risk of Bias tool for animal studies. ROBINS-I for clinical (non-randomized) studies. A custom quality-assessment checklist for in vitro studies (addressing sample preparation, testing methods, outcome standardization). Focus on synthesizing evidence across study types, identifying knowledge gaps, and informing clinical translation of liver-specific bioadhesives.
```

Study selection:
All identified records will be imported into a reference management program (e.g., Zotero) for duplicate removal. Two independent reviewers will then screen studies in two stages:

1. Title and abstract screening: Selection of potentially relevant articles.
2. Full-text assessment: Final decision on inclusion based on the full-text article.

Any disagreements between reviewers will be resolved by discussion or by involving a third reviewer. The selection process will be documented and presented as a PRISMA flow diagram.

Data extraction:
Two reviewers will independently extract data from included studies using a standardized form. An example data-extraction table is provided below:

Section	Data to be extracted
1. General information	Study ID; Author; Publication year; Country; DOI; Publication type (article, abstract); Funding source; Conflicts of interest
2. Methodology	Study design (in vitro, in vivo animal, clinical); Model (e.g., isolated porcine organ); Study aim; Hypothesis
3. Population / Model	Organ type; Animal species / human; Number of samples / animals / patients; Tissue characteristics (healthy, injured)

Section	Data to be extracted
4. Intervention	Adhesive name; Manufacturer; Primary biological component; Source (animal-derived, recombinant); Preparation method; Concentration; Application volume/mass; Polymerization / setting time
5. Comparison	Comparator method (sutures, another adhesive, placebo); Description of the comparator method
6. Primary outcomes	Sealing efficacy: Measurement method; Value (mean, SD); Units; Statistical significance. Adhesive strength: Strength type (tensile, shear); Measurement method; Value; Units; Statistical significance.
7. Secondary outcomes	Biocompatibility: Assessment method (histology, marker analysis); Results; Conclusions. Clinical outcomes: Complications, operative time, etc. (for clinical studies).
8. Conclusions and notes	Authors' main conclusions; Limitations noted by the authors; Reviewer comments

Risk of bias (study quality) assessment:
The quality of included studies will be assessed by two independent reviewers using the following tools:

- For preclinical animal studies: SYRCLE's Risk of Bias tool.
- For in vitro studies: a customized checklist may be developed based on existing guidance (e.g., QUADAS-2 for diagnostic accuracy studies, adapted for methodological quality assessment).
- For human studies (if identified): the Cochrane Risk of Bias tool for randomized trials or ROBINS-I for non-randomized studies.

Certainty of the body of evidence

The certainty (confidence) of evidence for each key outcome will be assessed by two independent reviewers using the GRADE (Grading of Recommendations, Assessment, Development and Evaluations) approach.

- For clinical studies (if identified): The standard GRADE approach will be used. Evidence from randomized controlled trials (RCTs) will initially be rated as high-certainty evidence but may be downgraded by one or more levels for the following reasons:
    1. Risk of bias: Based on assessments using Cochrane RoB 2 or ROBINS-I.
    2. Inconsistency: Unexplained heterogeneity or conflicting results.
    3. Imprecision: Wide confidence intervals crossing a threshold of clinical importance.
    4. Indirectness: Differences in population, interventions, comparators, or outcomes relative to the question of interest.
    5. Publication bias.
- For preclinical animal studies and in vitro studies: Because the standard GRADE framework was developed for clinical research, an adapted approach will be applied to appraise certainty of

preclinical evidence (e.g., SYRCLE's tool for assessing the certainty of evidence from animal studies or other accepted methods). The appraisal will likewise consider domains analogous to GRADE, including risk of bias, inconsistency, imprecision, and indirectness.

Findings will be presented in a summary table (e.g., a "Summary of Findings" table for clinical outcomes) and reflected in the narrative synthesis. The final certainty for each outcome will be classified as high, moderate, low, or very low.

Data synthesis:
Given the expected heterogeneity in study designs, adhesive types, and outcome assessment methods, a narrative (descriptive) synthesis will be performed. Results will be grouped by organ model, adhesive type, and outcome type and presented in tables and text.

If sufficient homogeneity is identified within a study subgroup (e.g., several studies comparing tensile strength of the same adhesive type on porcine liver), a meta-analysis will be considered. When interpreting both narrative synthesis and meta-analysis findings, the certainty of the body of evidence assessed using GRADE (or its adaptation for preclinical studies) will be taken into account.
