# Supplementary material for: Bio-Based Hemostatic Adhesives, Sealants, and Local Hemostatic Agents for Liver Tissue: A Systematic Review with Meta-Analysis of Preclinical Data
Source: J Funct Biomater. 2026 Oct 11;17(10):511. doi: 10.3390/jfb17100511 (PMC13706002; doi:10.3390/jfb17100511)
Supplement: Supplementary file 1 [file jfb-17-00511-s001.zip › S2_Search_strategies.pdf]

Search query for PubMed:

```
(

  liver[tiab] OR hepatic[tiab] OR hepatectom*[tiab]

)

AND

(

  adhesiv*[tiab] OR glue*[tiab] OR "biological glue"[tiab] OR "bio glue"[tiab] OR bioadhesive*[tiab] OR bioglue[tiab] OR "surgical sealant"[tiab] OR "surgical sealants"[tiab] OR "tissue sealant"[tiab] OR "tissue sealants"[tiab] OR "hemostatic agent"[tiab] OR "hemostatic agents"[tiab] OR "haemostatic agent"[tiab] OR "haemostatic agents"[tiab] OR "fibrin glue"[tiab] OR "fibrin sealant"[tiab] OR "fibrin adhesive"[tiab] OR gelatin[tiab] OR albumin[tiab] OR chitosan[tiab] OR alginate[tiab] OR collagen[tiab] OR hydrogel*[tiab] OR "crosslinked gelatin"[tiab] OR "natural adhesive"[tiab] OR "natural adhesives"[tiab] OR "protein adhesive"[tiab] OR "polysaccharide adhesive"[tiab] OR "mussel inspired"[tiab] OR "marine derived"[tiab] OR "plant derived"[tiab]

)

AND

(

  adhes*[tiab] OR bond*[tiab] OR seal*[tiab] OR hemosta*[tiab] OR haemosta*[tiab] OR strength*[tiab] OR tensil*[tiab] OR "shear strength"[tiab] OR "burst pressure"[tiab] OR "peel strength"[tiab] OR leak*[tiab] OR airtight*[tiab] OR watertight*[tiab] OR biocompatib*[tiab] OR cytotoxic*[tiab] OR degrad*[tiab] OR inflammat*[tiab] OR "tissue response"[tiab] OR "mechanical propert*"[tiab] OR "adhesive strength"[tiab] OR "bonding strength"[tiab] OR "time to hemostasis"[tiab] OR "bleeding time"[tiab] OR "blood loss"[tiab] OR "tissue healing"[tiab] OR "wound closure"[tiab] OR biocompatibility[tiab] OR biodegradation[tiab] OR "tissue regeneration"[tiab]

)

NOT

(

  cyanoacrylate*[tiab] OR "synthetic adhesive"[tiab] OR "synthetic adhesives"[tiab] OR "synthetic polymer"[tiab] OR "synthetic polymers"[tiab] OR polyurethane[tiab] OR "acrylic adhesive"[tiab] OR "acrylic adhesives"[tiab]

)

NOT

(

  kidney[tiab] OR renal[tiab] OR spleen[tiab] OR splenic[tiab] OR lung[tiab] OR pulmonary[tiab] OR pancreas[tiab] OR pancreatic[tiab] OR skin[tiab] OR cutaneous[tiab] OR dermal[tiab] OR dermis[tiab] OR epidermis[tiab] OR bone[tiab] OR cardiac[tiab] OR vascular[tiab] OR dental[tiab] OR orthodontic[tiab] OR endodontic[tiab] OR periodontal[tiab] OR tooth[tiab] OR teeth[tiab] OR "wound healing"[tiab] OR "wound repair"[tiab] OR scar[tiab] OR cicatrix[tiab] OR "burn wound"[tiab] OR "skin ulcer"[tiab] OR "pressure ulcer"[tiab] OR ulcer[tiab] OR "cutaneous wound"[tiab] OR keloid[tiab] OR "scar formation"[tiab] OR post-acne[tiab] OR "acne scars"[tiab]
```

)

Search query for Dimensions:

```
(

  liver OR hepatic OR hepatectomy

)

AND

(

  adhesive OR glue OR biological glue OR bio glue OR bioadhesive OR bioglue OR "surgical sealant" OR "tissue sealant" OR "hemostatic agent" OR "haemostatic agent" OR "fibrin glue" OR "fibrin sealant" OR "fibrin adhesive" OR gelatin OR albumin OR chitosan OR alginate OR collagen OR hydrogel OR "crosslinked gelatin" OR "natural adhesive" OR "protein adhesive" OR "polysaccharide adhesive" OR "mussel inspired" OR "marine derived" OR "plant derived"

)

AND

(

  adhesi OR bond OR seal OR hemosta OR haemosta OR strength OR tensile OR "shear strength" OR "burst pressure" OR "peel strength" OR leak OR airtight OR watertight OR biocompatib OR cytotox OR degrad OR inflammat OR "tissue response" OR "mechanical propert" OR "adhesive strength" OR "bonding strength" OR "time to hemostasis" OR "bleeding time" OR "blood loss" OR "tissue healing" OR "wound closure" OR biocompatibility OR biodegradation OR "tissue regeneration"

)

NOT

(

  cyanoacrylate OR "synthetic adhesive" OR "synthetic polymer" OR polyurethane OR "acrylic adhesive"

)

NOT

(

  kidney OR renal OR spleen OR splenic OR lung OR pulmonary OR pancreas OR pancreatic OR skin OR cutaneous OR dermal OR dermis OR epidermis OR bone OR cardiac OR vascular OR dental OR orthodontic OR endodontic OR periodontal OR tooth OR teeth OR "wound healing" OR "wound repair" OR scar OR cicatrix OR "burn wound" OR "skin ulcer" OR "pressure ulcer" OR ulcer OR "cutaneous wound" OR keloid OR "scar formation" OR post-acne OR "acne scars"

)
```

Search query for Consensus:

What is the effectiveness and safety of biological adhesives for achieving hemostasis and sealing liver tissues compared to standard surgical methods or other bio adhesives, according to in vitro, preclinical and clinical studies?

Search query for Scispace:

Act as a methodological lead and do a systematic literature review on "biological adhesives and sealants for liver tissue: efficacy, mechanical properties, and biocompatibility in preclinical and clinical studies" (no date restrictions). Search PubMed, Web of Science, Scopus, and Google Scholar comprehensively. Inclusion criteria: original studies (in vitro, animal, human) evaluating biological adhesives/sealants (e.g., fibrin-, gelatin-, collagen-, chitosan-, alginate-based, natural hydrogels, mussel-inspired, or plant-derived materials) applied to liver tissue. Outcomes of interest include hemostasis time, sealing/leak pressure, adhesive/tensile/shear strength, biocompatibility (inflammatory response, cytotoxicity), biodegradation, and clinical outcomes (e.g., postoperative complications, bile leak, blood loss). Exclusion criteria: synthetic adhesives (cyanoacrylates, polyurethanes, acrylics); studies focused on other organs (kidney, spleen, lung, pancreas, skin, bone, cardiovascular, dental, or general wound healing); non-research publications (reviews, editorials, case reports without original data). Deliver: A PRISMA flow diagram of the study selection process. Risk-of-bias tables using: SYRCLE’s Risk of Bias tool for animal studies. ROBINS-I for clinical (non-randomized) studies. A custom quality-assessment checklist for in vitro studies (addressing sample preparation, testing methods, outcome standardization). Focus on synthesizing evidence across study types, identifying knowledge gaps, and informing clinical translation of liver-specific bioadhesives.
