# Supplementary material for: Bio-Based Hemostatic Adhesives, Sealants, and Local Hemostatic Agents for Liver Tissue: A Systematic Review with Meta-Analysis of Preclinical Data
Source: J Funct Biomater. 2026 Oct 11;17(10):511. doi: 10.3390/jfb17100511 (PMC13706002; doi:10.3390/jfb17100511)
Supplement: Supplementary file 1 [file jfb-17-00511-s001.zip › Table_S1_Study-by-domain_SYRCLE_risk-of-bias_matrix_for_preclinical_in_vivo_animal_assessments.pdf]

Supplementary Table S1

Complete study-by-domain SYRCLE risk-of-bias matrix for preclinical/in vivo animal assessments included in the review evidence base (domain judgments: Low / Unclear / High). Human clinical studies and the veterinary RCT are summarized in main-text Table 15 (RoB 2 / ROBINS-I / adapted RoB 2). Two Jiang et al. (2023) reports are listed separately as [27] and [43]. Aggregate SYRCLE percentages remain as reported in main-text Table 3.

Study	Seq. generation	Baseline chars.	Alloc. conceal.	Random housing	Caregiver blind.	Random outcome	Assessor blind.	Incomplete data	Selective report.	Other bias	Overall
Anderson et al., 2025 [36] (veterinary RCT; also in Table 15)	Unclear	Low	Unclear	Unclear	High	Unclear	Unclear	Low	Low	Low	High/Unclear risk of performance bias
Baghdasarian et al., 2022 [9]	Unclear	Low	Unclear	Unclear	High	Unclear	Unclear	Low	Low	Low	High/Unclear risk of performance bias
Barsky et al., 2022 [37]	Unclear	Low	Unclear	Unclear	High	Unclear	Unclear	Low	Low	Low	High/Unclear risk of performance bias
Cao et al., 2019 [38]	Unclear	Low	Unclear	Unclear	High	Unclear	Unclear	Low	Low	Low	High/Unclear risk of performance bias
Deineka et al., 2021 [40]	Unclear	Low	Unclear	Unclear	High	Unclear	Unclear	Low	Low	Low	High/Unclear risk of performance bias
Zhang et al., 2020 [47]	Unclear	Low	Unclear	Unclear	High	Unclear	Unclear	Low	Low	Low	High/Unclear risk of performance bias
Freundlich et al., 2023 [28]	Unclear	Low	Unclear	Unclear	High	Unclear	Unclear	Low	Low	Low	High/Unclear risk of performance bias
Eskildsen et al., 2023 [16]	Unclear	Low	Unclear	Unclear	High	Unclear	Unclear	Low	Low	Low	High/Unclear risk of performance bias
Fontes et al., 2018 [41]	Unclear	Low	Unclear	Unclear	High	Unclear	Unclear	Low	Low	Low	High/Unclear risk of performance bias
Guo et al., 2021 [10]	Unclear	Low	Unclear	Unclear	High	Unclear	Unclear	Low	Low	Low	High/Unclear risk of performance bias
Guo et al., 2025 [42]	Unclear	Low	Unclear	Unclear	High	Unclear	Unclear	Low	Low	Low	High/Unclear risk of performance bias
Haghniaz et al., 2023 [29]	Unclear	Low	Unclear	Unclear	High	Unclear	Unclear	Low	Low	Low	High/Unclear risk of performance bias
Jain et al., 2025 [21]	Unclear	Low	Unclear	Unclear	High	Unclear	Unclear	Low	Low	Low	High/Unclear risk of performance bias
Jia et al., 2023 [24]	Unclear	Low	Unclear	Unclear	High	Unclear	Unclear	Low	Low	Low	High/Unclear risk of performance bias
Jiang et al., 2023 [27]	Unclear	Low	Unclear	Unclear	High	Unclear	Unclear	Low	Low	Low	High/Unclear risk of performance bias
Jiang et al., 2023 [43]	Unclear	Low	Unclear	Unclear	High	Unclear	Unclear	Low	Low	Low	High/Unclear risk of performance bias
Ko et al., 2025 [25]	Unclear	Low	Unclear	Unclear	High	Unclear	Unclear	Low	Low	Low	High/Unclear risk of performance bias
Lake et al., 2023 [2]	Unclear	Low	Unclear	Unclear	High	Unclear	Unclear	Low	Low	Low	High/Unclear risk of performance bias
Wang et al., 2022 [44]	Unclear	Low	Unclear	Unclear	High	Unclear	Unclear	Low	Low	Low	High/Unclear risk of

											performance bias
Liu et al., 2024 [30]	Unclear	Low	Unclear	Unclear	High	Unclear	Unclear	Low	Low	Low	High/Unclear risk of performance bias
Luo et al., 2024 [22]	Unclear	Low	Unclear	Unclear	High	Unclear	Unclear	Low	Low	Low	High/Unclear risk of performance bias
MacDonald et al., 2017 [1]	Unclear	Low	Unclear	Unclear	High	Unclear	Unclear	Low	Low	Low	High/Unclear risk of performance bias
Patterson et al., 2025 [5]	Unclear	Low	Unclear	Unclear	High	Unclear	Unclear	Low	Low	Low	High/Unclear risk of performance bias
Campos-Cuerva et al., 2019 [19]	Unclear	Low	Unclear	Unclear	High	Unclear	Unclear	Low	Low	Low	High/Unclear risk of performance bias
Ramot et al., 2021 [18]	Unclear	Low	Unclear	Unclear	High	Unclear	Unclear	Low	Low	Low	High/Unclear risk of performance bias
Slezak et al., 2018 [20]	Unclear	Low	Unclear	Unclear	High	Unclear	Unclear	Low	Low	Low	High/Unclear risk of performance bias
Sánchez Del Valle et al., 2025 [4]	Unclear	Low	Unclear	Unclear	High	Unclear	Unclear	Low	Low	Low	High/Unclear risk of performance bias
Wang et al., 2025 [45]	Unclear	Low	Unclear	Unclear	High	Unclear	Unclear	Low	Low	Low	High/Unclear risk of performance bias
Williams et al., 2023 [46]	Unclear	Low	Unclear	Unclear	High	Unclear	Unclear	Low	Low	Low	High/Unclear risk of performance bias
Du et al., 2024 [26]	Unclear	Low	Unclear	Unclear	High	Unclear	Unclear	Low	Low	Low	High/Unclear risk of performance bias
Xuan et al., 2023 [31]	Unclear	Low	Unclear	Unclear	High	Unclear	Unclear	Low	Low	Low	High/Unclear risk of performance bias
Xue et al., 2023 [32]	Unclear	Low	Unclear	Unclear	High	Unclear	Unclear	Low	Low	Low	High/Unclear risk of performance bias
Gao et al., 2023 [3]	Unclear	Low	Unclear	Unclear	High	Unclear	Unclear	Low	Low	Low	High/Unclear risk of performance bias
Zhang et al., 2023 [12]	Unclear	Low	Unclear	Unclear	High	Unclear	Unclear	Low	Low	Low	High/Unclear risk of performance bias
Zhao et al., 2024 [23]	Unclear	Low	Unclear	Unclear	High	Unclear	Unclear	Low	Low	Low	High/Unclear risk of performance bias
Zhu et al., 2022 [6]	Unclear	Low	Unclear	Unclear	High	Unclear	Unclear	Low	Low	Low	High/Unclear risk of performance bias
Zou et al., 2025 [48]	Unclear	Low	Unclear	Unclear	High	Unclear	Unclear	Low	Low	Low	High/Unclear risk of performance bias
Li et al., 2021 [11]	Unclear	Low	Unclear	Unclear	High	Unclear	Unclear	Low	Low	Low	High/Unclear risk of performance bias

Abbreviations: SYRCLE, Systematic Review Centre for Laboratory Animal Experimentation. Green = Low risk; yellow = Unclear risk; red = High risk.
